# A novel multiparameter sensor for shake flask cultivations: Online biomass, dissolved oxygen, and fluorescence monitoring for comprehensive bioprocess characterization

**DOI:** 10.1002/btpr.70035

**Published:** 2025-04-23

**Authors:** Lara Strehl, Anna‐Lena Kuhn, Kyra Hoffmann, Marcel Mann, Jørgen Barsett Magnus

**Affiliations:** ^1^ Aachener Verfahrenstechnik – Chair of Biochemical Engineering RWTH Aachen University Aachen Germany; ^2^ aquila biolabs GmbH Baesweiler Germany

**Keywords:** biomass, dissolved oxygen (DO), fluorescence, parallel online monitoring, shake flask

## Abstract

Shake flasks are one of the most widely used cultivation vessels in biotechnological process development. To improve the process understanding, new technologies have been reported for online monitoring of different parameters like oxygen, pH, or biomass in the last couple of years. However, most reports address the monitoring of a single parameter per shake flask. This work evaluates the ability to measure dissolved oxygen (DO), biomass, and fluorescence in parallel with a new Multiparameter Sensor (MPS). Therefore, abiotic tests for reproducibility, sensitivity, and accuracy were performed. In biological tests, different microbial systems were used to evaluate if a wide range of applications is feasible. This work demonstrates that three different parameters: DO, biomass, and fluorescence can be monitored online, in parallel, for various biological systems. The online data obtained provide crucial process knowledge, such as the start of intracellular product formation. Abiotic and biological tests showed good reproducibility, resolution, and sensitivity to changing environmental conditions. Compared to other existing measurement systems for DO or oxygen transfer rate, similar or in the former case, more data points can be recorded, allowing a detailed overview and a better understanding of the process.

**Abbreviations**
d_0_
: shaking diameter
DI water: deionized water
DO: dissolved oxygen
DOT: dissolved oxygen tension
GFP: green fluorescence protein
h: hour
L_O2_
: oxygen solubility (mol L^−1^ bar)
MES: 2‐(N‐morpholino)‐ethanesulfonic acid
min: minutes
MOPS: (N‐morpholino)‐propane sulfonic acid
MPS: Multiparameter Sensor
MTBE: methyl‐tert‐butyl ether
ms: milliseconds
*n*
: shaking frequency
OD_600_
: optical density at 600 nm
OTR: oxygen transfer rate
p_O2_^cal^
: oxygen partial pressure in gas phase during calibration (bar)
p_O2_^gas^
: oxygen partial pressure in gas phase (bar)
RAMOS: respiration activity monitoring system
rcf: relative centrifugal force
rep: replicates
rpm: rounds per minute
s: second
t: temperature
V_L_
: filling volume

## 1 INTRODUCTION

In academia and industry, shake flask cultivations are an essential part of biological research and process development for example, early strain characterization or finding the optimal medium composition.^1^ Although shake flasks are used extensively, online monitoring tools are few and far between. This results in extensive manual sampling or driving shake flask cultivations as black boxes, limiting the value of the experiments and increasing costs and time investments.

Within the last 20 years, new technologies have been developed to measure parameters online, closing this gap.^2^, ^3^, ^4^, ^5^, ^6^, ^7^ For monitoring the physiological state of an aerobic culture online, the oxygen transfer rate (OTR) and carbon dioxide transfer rate (CTR) can be measured via the commercially available RAMOS device (HiTec Zang GmbH, Herzogenrath, Germany) or the TOM device (Adolf Kühner AG, Birsfelden, Switzerland).^2^ An alternative approach involves measuring the dissolved oxygen (DO) in the liquid phase. Several studies have been conducted to determine this parameter online. The online measurement can be carried out using different types of chemosensors, like spots, patches, or nanoparticles, coated with a luminescent color sensitive to oxygen. The chemosensor underlies the principle of quenching. The sensor spots are attached to the inner part of the shake flask, while the nanoparticles are suspended in the liquid. The readout is facilitated from the outside of the shake flask via an optical fiber or coaster.^8^, ^9^, ^10^ Commercial systems for measuring the DO or dissolved oxygen tension (DOT) are available from PyroScience or PreSens.^11^, ^12^ However, the sensor spots are fixed to a position inside the shake flask, meaning that they are not in continuous contact with the bulk liquid. This configuration has been shown to result in problems, as described by Hansen et al. and Flitsch et al.^3^, ^4^ The optical sensor is also located at a fixed position when using nanoparticles. However, as the particles move in the bulk liquid, the position of the liquid can be tracked, and thus, the measurement is only triggered when the liquid is in front of the measuring window of the optical sensor.^3^, ^13^ To avoid the issue of a mixed signal, a higher filling volume and lower shaking frequency can be employed, thereby ensuring that the sensor spot remains continuously covered by the bulk liquid. The quantification of biomass online can be accomplished through the utilization of non‐invasive optical techniques, such as the measurement of scattered light or backscatter in shake flasks.^14^, ^15^, ^16^ Systems for monitoring biomass online are available from sbi (Scientific Bioprocessing) with the CGQ and PreSens (Precision Sensing GmbH) with the SFR vario.^11^, ^17^

Online fluorescence measurement is so far only established at the microtiter plate scale.^18^, ^19^, ^20^ While parallel online monitoring of several parameters is common for bioreactor scales, this is still rare for shake flasks. Compared to bioreactors, the options for shake flasks are often limited to one parameter per device. To unlock the full potential of easy‐to‐set‐up shake flask cultivations, online monitoring is crucial. Schneider et al. could show in their work a combination of pH and DOT measurement with the commercially available SFR vario, where oxygen sensor spots were used (PreSens Precision Sensing GmbH).^21^ Dinter et al. showed the ability of online monitoring of DOT, pH, biomass, and viscosity in parallel by using Oxnano nanoparticles from PyroScience GmbH with a new ShakeVisc module.^22^ In contrast to the equipment used in this work, this module is not available commercially. In this study, the potential of parallel biomass, dissolved oxygen (DO), and fluorescence measurements with the Multiparameter Sensor (MPS) and DO Sensor Pill was evaluated (Aquila Biolabs GmbH, Scientific Bioprocessing INC). The MPS is a sensor plate that is positioned in an adapter below the shake flask and can measure biomass, DO, and fluorescence via various integrated measuring modules. Dissolved oxygen was measured using the quenching effect with the DO Sensor Pill, which is coated with a chemosensor. The pill rotates with the bulk liquid in the shake flask. Abiotic tests were performed to verify accurate and sensitive results from the DO Sensor Pill to changing oxygen concentrations in the liquid. The biomass and fluorescence can be measured at different wavelengths and emission lengths. To show a wide range of applications, cultivations with the following microorganisms were performed: *Escherichia coli, Corynebacterium glutamicum, Komagataella phaffii, and Ustilago maydis*.

*E. coli* and *C. glutamicum* are used in a wide range of industrial applications to produce for example, drugs or food and cosmetic ingredients.^23^, ^24^, ^25^, ^26^ They have in common being fast‐growing organisms with high oxygen consumption.^23^, ^26^ Here, they were used to investigate the sensitivity of the DO measurement with the MPS. To demonstrate a broad range of applications for the system, the growth of the yeast *K. phaffii* and the dimorphic fungi *U. maydis* was analyzed. Moreover, the used *K*. *phaffii* strain produces a GFP protein, which should be tracked with the MPS. As the selected *U*. *maydis* strain produces triglycerides intracellularly, this study investigated whether the product affects DO and biomass measurement.

## 2 MATERIALS AND METHODS

Cultivations were run with the Multiparameter Sensor (MPS) and Dissolved Oxygen (DO) Sensor Pills (Scientific Bioprocessing, INC, Pittsburgh, USA and aquila biolbas GmbH, Baesweiler, Germany), the RAMOS device^2^, ^27^ and oxygen sensor spots (PyroScience GmbH, Aachen, Germany). Experiments with the different measuring systems and offline flasks for sampling were carried out in the same incubator (Climo‐Shaker ISF1‐X, Kühner, Birsfelden, Switzerland) (Figure S1). Different conditions, like the shaking frequency (*n*), the shaking diameter (d_0_), and temperature (t) were adjusted, depending on the experiment. The initial OD_600_ is specified for the respective experiment in the results section. Duplicates or triplicates were run in parallel. In Table 1, the different measurement systems, the provided online data, as well as the measurement principles are shown.

**TABLE 1:** Overview of different measurement systems used, the provided online data, the measurement principle, and the frequency of measurement points.

Measurement system	Online data provided	Measurement principle	Frequency of measurement points
MPS and dissolved oxygen (DO) Sensor Pill	DO, biomass, fluorescence	Luminescence dye, backscatter, emission	Every 30 seconds
RAMOS	Oxygen transfer rate (OTR)	Pressure in headspace	Every 30 minutes
Oxygen sensor spot	Dissolved oxygen tension (DOT)	Luminescence dye	Every second

### 2.1 Multiparameter Sensor (MPS) and dissolved oxygen (DO) Sensor Pill

**FIGURE 1:**
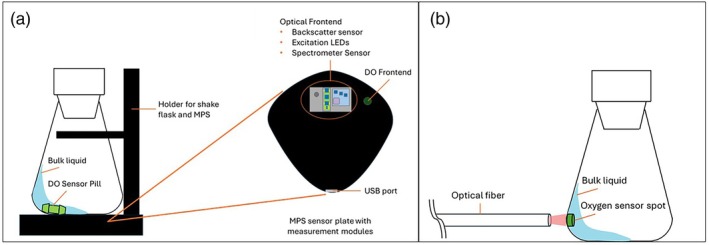
Schematic illustration and setup of the DO Sensor Pill measurement with the Multiparameter Sensor (MPS) module (a) and the DO sensor spot (b). The setup of the RAMOS device can be found elsewhere.^2^, ^27^, ^29^

The MPS is a device enabling the parallel measurement of biomass, DO, and fluorescence. The sensor is positioned below the shake flask in a special adapter (Figure 1a). The adapter ensures the correct positioning of the shake flask upon the measurement modules. Various measuring modules are installed in the MPS for parallel measurement of the cultivation parameters biomass, fluorescence, and DO (Figure 1a).^28^ The DO measurement is based on a platform using a DO Sensor Pill, which is coated with a luminescent dye sensitive to oxygen (further information on the DO Sensor Pill can be found in the ‘MPS User Guide’ provided by aquila biolabs GmbH and Scientific Bioprocessing INC^28^ and in the Data S1). While shaking, the DO Sensor Pill is moving with the in‐phase liquid and passing over the DO frontend of the MPS. The sensor emits a red light (610–630 nm), exciting the DO Sensor Pill, showing luminescence in the near‐infrared light region (NIR, 760–790 nm) due to the DO Sensor Pill dye. The luminescence depends on the level of oxygen present in the cultivation. The more oxygen is present in the cultivation liquid, the more the luminescence is quenched. Those changes in the luminescence are detected by the MPS. The biomass measurement is based on the backscattering principle. An LED can emit light in 12 different wavelengths,^28^ which is scattered back by the cells in the medium and detected by a photodiode. Thus, the signal increases with increasing number of cells, and a growth curve is generated. For the fluorescence measurement, the same LEDs can be used to excite the fluorophore as for the backscatter measurement, whereas the emission is detected using a spectrometer. In the case of an unknown fluorophore for which a characterization has not yet been established, a screening may be performed. The excitation/emission spectrum in its entirety can be covered, with all emission wavelengths being able to be combined with the excitation wavelengths.

The DO, biomass (backscatter), and fluorescence measurements are combined in a single system, with measurements triggered sequentially. Different measurement intervals can be set, with the minimum defined as the interval between wavelengths (e.g., 622, 645, and 940 nm for biomass at 30‐s intervals). If multiple measurements are configured for the same sensor, the interval extends. Acquisition time refers to the duration the measurement is conducted, with the liquid in the shake flask illuminated during this period. The system issues an error if the minimum intervals are not met due to excessive parameters or high acquisition times. The spectrometer integration time for fluorescence can also be set, defining the light introduction duration. The MPS records as many sequences as possible within the acquisition time. The 100 ms window is optimal for GFP production, but different integration times may be better for fluorophores with higher excitation/emission wavelengths and lower concentrations.

The influence of the DO Sensor Pill regarding shear stress was analyzed beforehand via microscopy. No influence on the morphology of the cells could be seen (Figure S2).

### 2.2 Respiratory activity monitoring system (RAMOS)

With the in‐house built respiration activity monitoring system (RAMOS), it is possible to measure the oxygen transfer rate (OTR). Commercial versions of the RAMOS device can be acquired from HiTec Zang GmbH (Herzogenrath, Germany) or Kühner AG (Birsfelden, Switzerland). Modified 250 mL shake flasks were used, which had a similar geometry to the other shake flasks used in this work (see also References 27, 29). For better comparability of results from the different measurement devices, the DO was calculated based on Flitch et al.^3^ (Equation S1).

### 2.3 Oxygen sensor spots

To measure the DO online with oxygen sensor spots, the Optical Oxygen Meter “FireStingO2” was used in combination with the self‐adhesive oxygen sensor spots (OXSP5‐ADH), an optical fiber with lens (SPFIB‐LNS) and a basic spot adapter (SPADBAS) (PyroScience GmbH, Aachen, Germany). A 250 mL shake flask was used for cultivation and the Pyro Workbench V1.5.0 software for data recording. In the following work, this DOT (dissolved oxygen tension) measuring system is referred to as oxygen sensor spot (Figure 1b).

### 2.4 Microorganisms and media

The biological experiments were conducted with four different strains: *Escherichia coli* BL21 DE3, *Corynebacterium glutamicum* DM 1933, *Ustilago maydis* MB215Δcyp1Δemt1, deposited at DSM17147 as MB215cyp1emt1,^30^ and *Komagataella phaffii* Mut^S^ host strain BSYBG11 (BG11) obtained from Bisy GmbH (Hofstaetten a. d. Raab, Austria). *K*. *phaffi*i contained a genomic integration of green fluorescent protein (GFP) expression cassette under the pCAT promoter. The cryo‐cultures of all strains were stored at −80°C.

In this work, pre‐ and main cultivation of *E. coli* were performed in Wilms‐MOPS medium.^31^ The composition of the main solution, thiamin solution, MgSO_4_ solution, and trace element solution can be found in the (Table S1). The main solution was autoclaved after the pH was adjusted to 7.5 by using 5 M NaOH. Directly before the medium was used, the thiamine, MgSO_4_, and trace element solutions were added to the main solution.

*C. glutamicum* DM 1933 was pre‐cultivated in YPG complex medium. The main cultivation was conducted in the modified CG‐XII medium^32^, ^33^ (Table S2). The trace element solution was adjusted to pH 1 with H_2_SO_4_. These solutions as well as the MOPS solution and urea solution (CO(NH_2_)_2_) were prepared and autoclaved separately. The individual media components were put together right before use. The pH was adjusted to 7.25 with 5 M NaOH.

For the pre‐ and main cultivation of *K*. *phaffii* Mut^S^ (host strain BSYBG11 (BG11)) obtained from Bisy GmbH (Hofstaetten a. d. Raab, Austria, Genomic integration of GFP protein expression cassette under pCAT) Syn6‐MES medium was used^34^ (Table S3). The pH of the basic medium was adjusted to 6.0 by using 1 M NaOH. The ammonium sulfate solution was prepared separately. Directly before use, 300 mL/L of the basic medium, 20 mL/L of the (NH_4_)_2_SO_4_ solution, 10 mL/L of a sterile CaCl_2_ solution, 10 mL/L of the microelement stock solution, 10 mL/L of the vitamin stock solution, 10 mL/L of the trace element stock solution, and 20 mL/L of a glucose solution were added. To fill up the missing volume, sterilized DI water was used. The two vitamin solutions were mixed after dissolving each separately. For induction of the main culture, 2% (v/v) methanol was added to the medium directly before use. After combining the different components of the medium, no pH adjustment was required.

For the pre‐ and main cultivation of *U*. *maydis*, the modified Verduyn mineral medium was used with different carbon‐to‐nitrogen ratios^35^ (Table S4). All solutions were combined directly before cultivation. For the pre‐cultivation, the Verduyn medium was used, with a lower carbon‐to‐nitrogen ratio. For the main cultivation with triglyceride production, an increased carbon‐to‐nitrogen ratio was used, as a nitrogen limitation triggers triglyceride production. The starting pH of the medium was adjusted to 6.5 to prevent a strong pH drop caused by the produced triglycerides.

### 2.5 Cultivation conditions

Cultivations with the MPS, Oxygen Sensor Spots, and offline sampling were conducted in 250 mL unbaffled glass shake flasks (ROTILABO®, borosilicate glass 3.3, ROTH SELECTION, Carl Roth GmbH + Co. KG).

For the *E. coli* preculture, a completed Wilms‐MOPS medium with a glucose concentration of 20 g/L was inoculated with a cryo‐stock to a starting OD_600_ of 0.1. Subsequently, the preculture from the exponential growth phase was used to inoculate the main culture. For that, Wilms‐MOPS medium with a glucose concentration of 10 g/L was inoculated with a starting OD_600_ of 0.5. The main culture was transferred into the shake flasks with a filling volume of 4–10%, depending on the experiment, and incubated at 37°C at different shaking frequencies and diameters (250 and 350 rpm and 25 and 50 mm).

*C. glutamicum* was precultured in complex media with 20 g/L glucose and inoculated with a cryo‐stock to an OD_600_ of 0.1. The preculture was harvested via centrifugation at 2800 rcf for 10 min at the exponential growth phase to inoculate the main culture. For that, the minimal CG‐XII medium with a glucose concentration of 20 g/L was inoculated to a starting O_600_ of 0.1. The main culture was transferred into the shake flasks with a filling volume of 10% and incubated at 30°C at a shaking frequency of 250 rpm and a shaking diameter of 50 mm.

For the *K*. *phaffii* preculture, the completed Syn6‐MES medium without methanol and a glucose concentration of 10 g/L was inoculated to a starting OD_600_ of 0.1 from a cryo‐stock. The filling volume was 10%, the shaking frequency was set to 300 rpm with a shaking diameter of 50 mm, and the cultivation temperature was 30°C. The preculture was harvested at the exponential growth phase to inoculate the main culture. For the main culture, completed Syn6‐MES medium including 2% (v/v) methanol was used with a glucose concentration of 10 g/L. The starting OD_600_ was set to 0.2. The main culture was transferred into the shake flasks with a filling volume of 10% and incubated at 30°C at a shaking frequency of 250 rpm and a shaking diameter of 50 mm.

*U. maydis* precultures were done in the Verduyn medium with a lower carbon‐to‐nitrogen ratio (Table S4). The preculture was inoculated to an OD_600_ of 0.1 and harvested at the exponential growth phase. After harvesting, the preculture was washed with 0.9% (w/v) NaCl to avoid transferring nitrogen components into the main cultivation. This was done only in the experiments with *U*. *maydis*, as the strain only produces triglycerides under nitrogen limitation. For the main culture, the Verduyn medium with a higher carbon‐to‐nitrogen ratio was used to produce the desired triglycerides. The main culture was inoculated to an OD_600_ of 0.2 and transferred into the shake flasks with a filling volume of 10% and incubated at 30°C at a shaking frequency of 250 rpm and a shaking diameter of 50 mm.

### 2.6 Offline analysis

Offline samples were taken from offline reference shake flasks. Those shake flasks were filled with the same master mix of main culture as the online shake flasks. Offline and online shake flasks were incubated in the same incubator (Climo‐Shaker ISF1‐X, Kühner, Birsfelden, Switzerland). Offline samples from the online shake flasks were only taken after the cultivation was terminated. The sample frequency was based on the continuously shown online data.

The OD_600_ was measured photometrically at 600 nm in disposable cuvettes (UV cuvettes, semi‐micro, Brand, Wertheim, Germany) with a spectrophotometer (Genesys 202, Thermo Scientific, Darmstadt, Germany). If it was necessary, samples were diluted with 0.9% (w/v) NaCl to an OD_600_ of 0.1–0.4. The pH was measured with a pH meter (HI2211 pH/ORP meter, HANNA Instruments, Woonsocket, USA).

HPLC analyses were performed for glucose, methanol, acetate, l‐lysine, succinate, lactate, and formate with a Thermo Fisher Ultimate 3000 (Thermo Fisher Scientific, Waltham, Massachusetts, USA). To prepare the samples, the fermentation broth was centrifuged at 14000 rcf for 10 min and subsequently sterile filtrated. A Rezex ROA‐Organic Acid H + (8%) LC Column 300 × 7.8 (Phenomenex, Inc., Torrance (CA); USA) was used. The mobile phase was a 25 mM H_2_SO_4_ solution with a flow rate of 0.8 mL/min, at a column temperature of 75°C.

For the determination of the cell dry weight, 2 mL dried sample tubes were weighed with the precision scale (VWR, LA254i). Afterward, they were filled with 2 mL of the cultivation broth and centrifuged for 10 min at 14000 rcf. The supernatant was discarded, and the pellet was dried for approx. 24 h at 60°C in a universal oven (UN55, Memmert GmbH + Co. KG, Schwabach, Germany). The samples were weighed again, and the difference between the empty value and the filled value was calculated.

Triglyceride quantification for the experiments with *U. maydis* was done according to Matyash et al.^36^ 2.4 mL cultivation broth was disrupted via sonification with a Fisherbrand™ Model 120 Dismembrator with 1/8″ Microtip (Fisher scientific, Schwerte, Germany). From the disrupted samples, 2 mL were transferred to a 15 mL reaction tube, and 1.5 mL methanol and 5 mL methyl‐tert‐butyl ether (MTBE) were added. The falcons were shaken in a tabletop shaker ThermoMixer™ C (Fisher scientific, Schwerte, Germany) at 37°C for 30 min at 800 rpm. Afterward, 1.5 mL DI water was added and shaken for another 10 min. The samples were centrifuged for 10 min at 1000 rcf. The upper phase was collected into three HPLC vials. The HPLC vials were weighed before filling and after the evaporation of the MTBE.

To determine GFP fluorescence, a Multi‐Detection Microplate Reader (Synergy 4; BioTek, Winooski, VT, USA) was used. Therefore, 100 μL of the cultivation broth was directly transferred to a 96‐well flat bottom plate in triplicates. The medium was used as the reference blank. The excitation wavelength was set to 488 nm, and the emission wavelength to 520 nm. If necessary, the samples were diluted with 0.9% (m/v) NaCl.

### 2.7 Abiotic tests with gas mixing battery

Abiotic tests without microbial cells were performed in 250 mL RAMOS shake flasks with a filling volume of 10%. The RAMOS shake flasks can be gassed in a controlled manner, allowing different gas compositions to be set in the flasks. Sterile Wilms‐MOPS medium was used, and a DO Sensor Pill was added. The shake flasks were mounted on the MPS and connected with the gas in‐ and outlet of the RAMOS system. A shaking frequency of 250 rpm was set at a temperature of 30°C. Different oxygen concentrations were set in the flask to perform the sensitivity analysis. For this purpose, a gas mixing battery (built in‐house) was connected to the RAMOS system to generate different concentrations of oxygen. The gas mixing battery consists of a digital display and adjustment unit that controls two BROOKS 4800 series and a BROOKS GF40 mass flow controller (BROOKS INSTRUMENTS, Inc., Hatfield, Pennsylvania, USA). Compressed air and nitrogen were connected to the gas‐mixing battery. The respective flow rate for the two gases was set via the digital display, making it possible to set different oxygen concentrations in the flask and, therefore, also in the medium. The flow rate per flask was set to 69 mL/min.

## 3 RESULTS AND DISCUSSION

### 3.1 Abiotic tests for sensitivity and reproducibility

**FIGURE 2:**
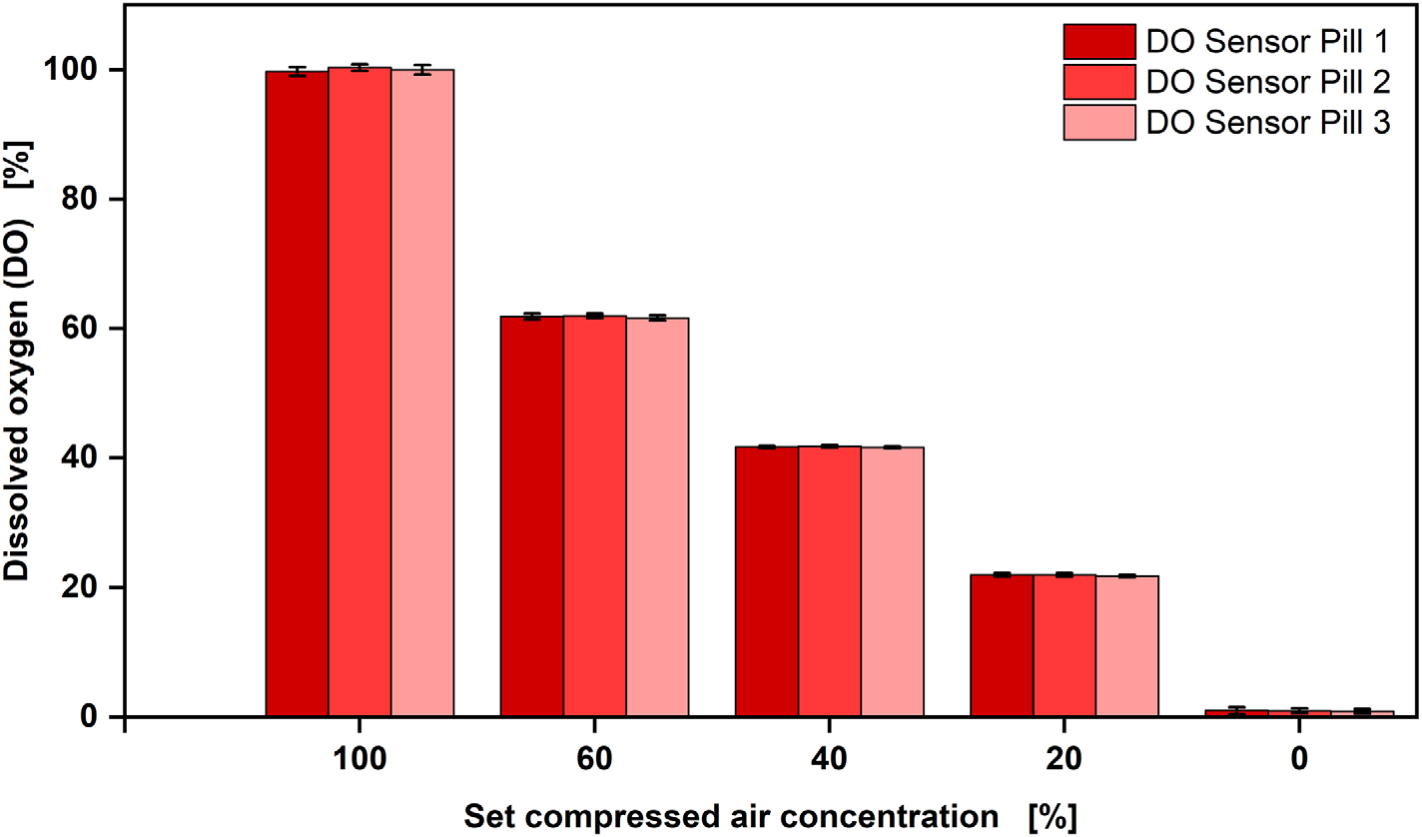
Sensitivity test with DO Sensor Pill in abiotic Wilms‐MOPS medium, gas mixture of compressed air and nitrogen with gas mixing battery. The RAMOS system was used for gassing, *n* = 250 rpm, d_0_ = 50 mm, t = 30°C, V_L_ = 25 mL, rep. = 3. Setting compressed air concentrations to 100%, 60%, 40%, 20%, and 0%.

The aim was to investigate the sensitivity and reproducibility of the DO Sensor Pill. For this purpose, different oxygen concentrations were set in the Wilms‐MOPS medium (abiotic) without microbial cells, using a gas mixing battery. At the same time, the data was recorded continuously to record changes (Figure 2).

The following settings were made: A mixture of N_2_ and compressed air with 100%, 60%, 40%, 20%, and 0% compressed air. At 100% compressed air, the DO Sensor Pill displays 100% dissolved oxygen. This is due to the calibration of the DO Sensor Pill, which was carried out using compressed air with an oxygen content of 21.95%. This value was set as 100% DO for the DO Sensor Pill. All set oxygen concentrations could be accurately measured by the three sensors with deviations in a range of 0.15–1.3%. A defined equilibration time was necessary to ensure that the set oxygen concentrations were achieved in both the liquid and the headspace of the shake flasks, since the different gas compositions were set one after another. The equilibration time depends on the shake flask volume, the volume of the headspace, and air flow, and was, in this case, 1.5 h. This could also be seen for the other oxygen concentrations. However, slight deviations could also be due to the gas mixing battery, which has limits in its accuracy. For this reason, a further test was carried out using a gas trap with a precisely defined gas composition. The DO Sensor Pill displayed the corresponding value with a deviation of 0.33%, maintaining this value over 120 h (Figure S3). This shows that the DO Sensor Pill delivers reproducible results and is sensitive and accurate at different dissolved oxygen concentrations. In addition, the measurement is robust over 120 h and thus suitable for longer cultivation periods.

### 3.2 Comparison with other existing measuring systems for online OTR and online DO measurements

**FIGURE 3:**
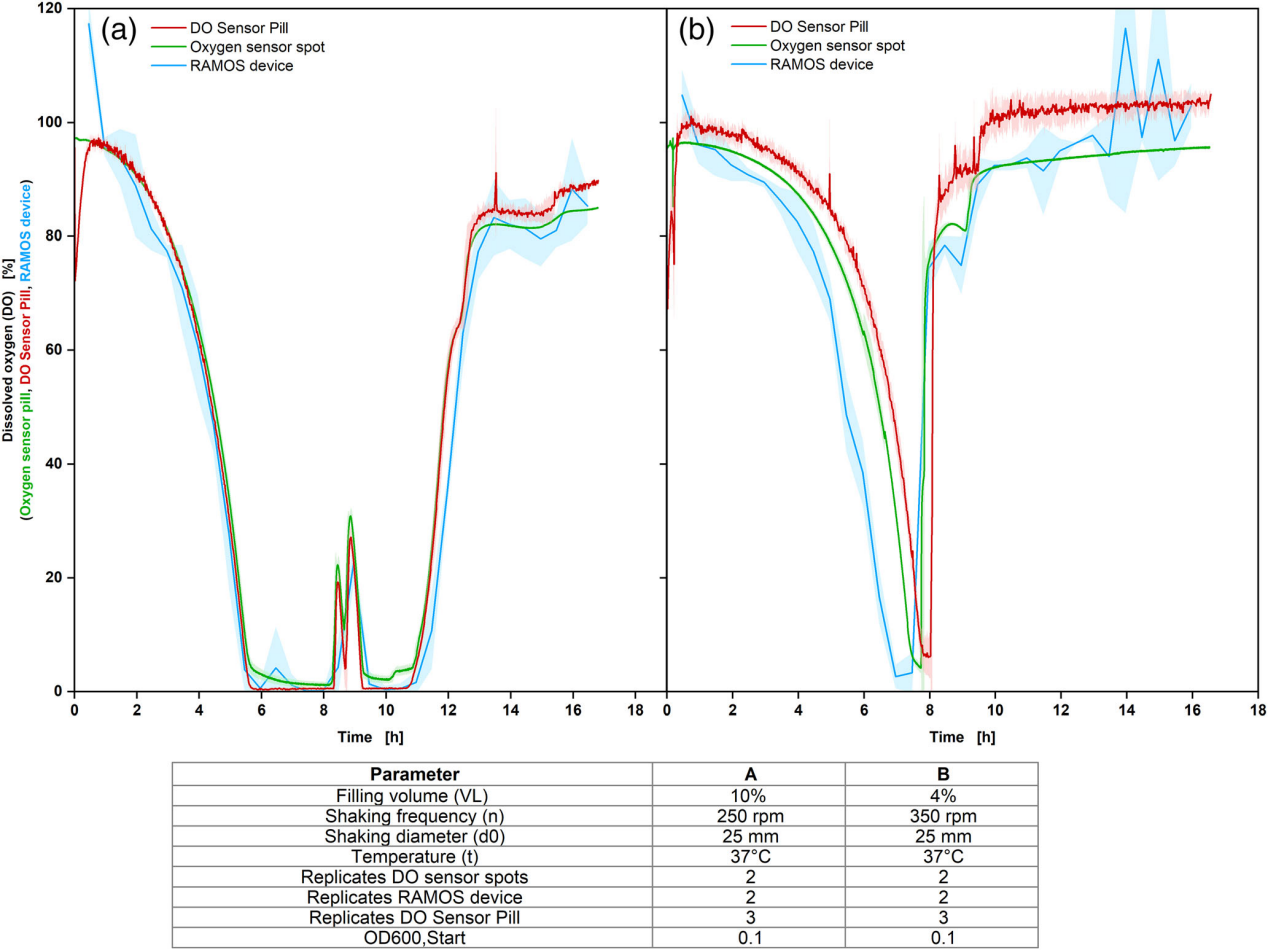
Comparison of dissolved oxygen (DO) measurements from the measuring systems: DO Sensor Pill, oxygen sensor spot, and RAMOS device with *Escherichia coli* BL 21 DE 3. Wilms‐MOPS medium; shadows indicate the standard deviations.

The DO Sensor Pill measurement was tested with two well‐established measurement systems to measure the dissolved oxygen content in liquids or the oxygen transfer rate (OTR). The chosen systems were the optical oxygen sensor method with the PyroScience equipment (oxygen sensor spot) and the RAMOS measurement system. The biological system selected was *E. coli*, cultivated under two different conditions, which differ in the filling volume, the shaking frequency, and the shaking diameter, commonly represented in the literature (Figure 3).^37^, ^38^, ^39^

The validity of these results was established by conducting the t‐tests. The *p*‐value obtained for the cultivation depicted in Figure 3a was 4.6 × 10^−5^. For Figure 3b, a remarkably more significant *p*‐value of 2.25 × 10^−17^ was recorded. These results indicate a high degree of comparability between the data from the DO Sensor Pill and the DO sensor spots.

The DO Sensor Pill shows a higher noise level at high DO ranges. This was already stated in the work of Tolosa et al.^9^ and can be due to a non‐linear calibration plot of oxygen concentration and emitted intensity caused by the inherent property of the dye of the DO Sensor Pill.^40^, ^41^ Compared to the displayed calculated DO values of the RAMOS device, the oxygen sensor spot and the DO Sensor Pill provide shorter sampling intervals, resulting in a higher resolution of the DO measurement compared to the RAMOS measurement (Figure S4). This issue can be lessened to some extent by decreasing the measurement interval of the RAMOS device.

Using the DO Sensor Pill, small spikes were detected in both tests (for example, at 5 h Figure 3b or 13.5 h Figure 3a). One reason could be that the DO Sensor Pill was not completely covered with medium for a short amount of time. This assumption could be confirmed by the higher amounts of peaks at lower filling volumes (Figure 3b). A time offset in the online measurement data (Figure 3b) resulted from flasks being placed in the shaker at different times due to calibration and pressure tests. To ensure biological comparability, a time offset at inoculation was avoided. However, a repeated test with only the RAMOS device and DO Sensor Pill showed no delay, indicating the handling issue was the main cause (Figure S5). An offset was observed in the dissolved oxygen (DO) levels, with the DO Sensor Pill showing higher DO content, especially after 9 h of cultivation. The discrepancy between RAMOS data and DO values may be due to variations in the OTR‐to‐DO conversion. The deviation from sensor spots is likely due to calibration, as seen with the initial 96% DO at the start. Additionally, the low filling volume of the DO Sensor Pill (4%) may have caused oxygen content measurements in the headspace. A filling volume of 5% is recommended as the minimal filling volume, showing more comparable results regarding the DO level (Figure S6).^28^ For this reason, it is important to ensure that a sufficient filling volume is chosen. In accordance with the information supplied by the manufacturer, a filling volume of 10% for a 250 mL shake flask should be employed. This condition is predominantly selected in industry applications and was consequently selected for subsequent experiments. Notably, the DO Sensor Pill measured a DO level around 0% in the time range of 5.5–10.5 h, whereas the oxygen sensor spots were around 3% (Figure 3a). Since an oxygen limitation was measured by RAMOS, too, a DO very close to zero was also expected for the oxygen sensor spots. This phenomenon was already observed by Flitch et al.^3^ and Hansen et al.^4^ and is attributed to the fixed position of the sensor spots resulting in a mixed signal between the DO in the bulk liquid and the oxygen in the headspace of the shake flask. At the beginning of the measurement with the DO Sensor Pill, the DO started at approx. 80% in both experiments. This could be attributed to the temperature equilibration of the medium after starting the shaker (Figures S7 and S8). Another assumption would be that the pill with the dye has to adapt to the liquid and the environmental conditions. In comparison, the oxygen sensor spots were left to swell for 24 h in sterile water for preparation.

Offline samples were taken at the end of each cultivation for each online measurement system obtained, revealing no statistically significant differences. Furthermore, it was demonstrated that neither the MPS nor the DO Sensor Pill exhibited any toxic effects on the cells (Figure S9).

### 3.3 Combination of online DO and online biomass measurement with the MPS and the DO Sensor Pill

**FIGURE 4:**
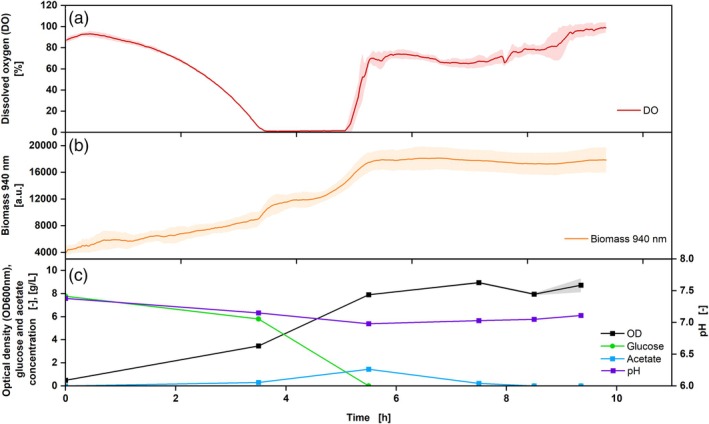
Cultivation of *Escherichia coli* BL 21 DE 3, Wilms MOPS medium, DO Sensor Pill (a, red), and biomass measurement (b, orange) with offline sampling OD_600_ (c, black), glucose (c, green), acetate (c, blue) and pH (c, purple), V_L_ = 10%, *n* = 350 rpm, d_0_ = 50 mm, t = 37°C, rep. = 3, OD_600,Start_ = 0.5. Shadows indicate the standard deviations.

**FIGURE 5:**
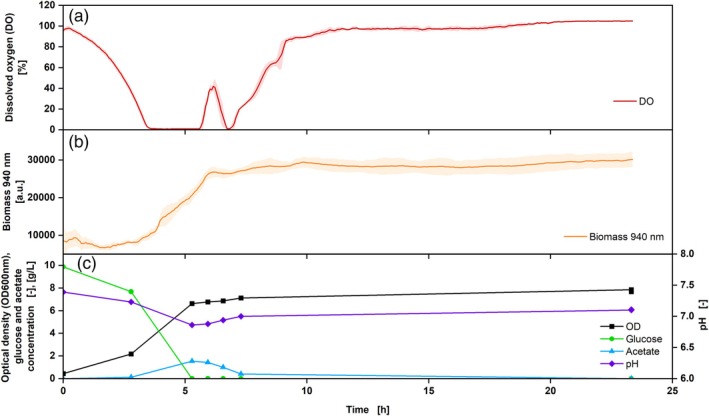
Cultivation of *Escherichia coli* BL 21 DE 3, Wilms‐MOPS medium, DO Sensor Pill, DO (a, red) and biomass measurement at 940 nm (b, orange) with offline sampling: OD_600nm_ (c, black), glucose (c, green), acetate (c, blue) and pH (c, purple), V_L_ = 10%, *n* = 250 rpm, d_0_ = 50 mm, t = 37°C, rep. = 3, OD_600_,_Start_ = 0.5. Shadows indicate the standard deviations.

In addition to the DO measurement using the DO Sensor Pill, the biomass progression over the fermentation time can be measured online using the MPS. To ensure parallel measurements of biomass and DO, measurements were carried out with *E. coli* under two different shaking frequencies (shaking diameter of 50 mm, 350 and 250 rpm shown in Figures 4 and 5 respectively).

The experimental approach with a shaking frequency of 350 rpm shows a decreasing DO in the first 3.5 h, forming a plateau of around 1% thereafter, which lasted for over an hour (Figure 4), indicating an oxygen limitation.^2^ This was also seen in the RAMOS data, which were run in parallel to the shake flasks with the DO Sensor Pill (Figure S10). Subsequently, after the glucose was consumed (5 h), the DO rose again. The increase in biomass was measured via the scattered light intensity at a wavelength of 940 nm. From the time of oxygen limitation (from 3.5 to 5 h), a plateau in the measurement was seen, which may indicate a slower increase in biomass under oxygen‐limiting conditions.^42^ This was not recognizable in the offline OD_600_ data since fewer samples were taken. However, the different growth stages of the culture (e.g., exponential or stationary phase) correspond between online and offline data. Based on the HPLC data, it can be observed that glucose was consumed after approx. 5.5 h. During oxygen limitation, the production of acetate increased more strongly, which is also indicated by the decreasing pH. After the glucose has been consumed, the acetate was also metabolized, resulting in an increase in the pH. As soon as this was also consumed, the DO rose again to 100% (Figure 5).

The experimental approach with a shaking frequency of 250 rpm shows that the triplicates of the DO Sensor Pill exhibit the same DO course during fermentation and are, therefore, reproducible (Figure 5). These results also agree with the RAMOS measurement (Figure S11), where an oxygen limitation can be recognized for more than 2 h. Compared to the experiment described above (Figure 4), the oxygen limitation was prolonged, which can be explained by the lower shaking frequency. The increased shaking frequency creates a larger exchange surface between the medium and the ambient air, which favors the transfer of oxygen into the medium.^43^ A prolonged oxygen limitation results in a longer process due to reduced respiration of the cells.^44^ In this experiment, the online biomass signal matches the offline OD_600_ values. A discrepancy in online and offline measurements was only observed regarding the time at which the plateau of the biomass concentration was reached. While the online measured biomass concentration increased during the first 6 h of cultivation, the offline data already stated a plateau after 5 h. The DO value displays an oxygen concentration of approximately 0% from 3.6 to 6 h, indicating that the cells maintain respiratory activity. The demonstration of respiratory activity indicates, among others, the increase in biomass concentration. Consequently, the DO measurements verify the biomass concentration measurements obtained online. In the offline samples, no significant differences can be observed between the OD samples of 5 and 6 h. This discrepancy may be attributable to inaccuracies in the manual sampling procedure. This finding emphasizes the importance of continuous online data measurement, as such influencing factors can be avoided. Furthermore, the correlation between offline and online biomass measurement was demonstrated in a correlation plot (Figure S12). Conclusively, until the termination of cultivation, both online and offline measurements demonstrate a slight increase in biomass concentration. The offline samples also show that the glucose was used up after around 5.5 h, which is why the DO briefly rose again. However, the metabolism of the produced acetate during oxygen limitation causes it to fall again, and only when both acetate and glucose are completely metabolized, does the DO rise again.

These two tests show that under both shaking conditions, an online biomass measurement can be carried out in parallel to the online DO measurement without the need for additional equipment.

In addition, it was investigated whether the online DO measurement with the DO Sensor Pill and the MPS, as well as the online biomass measurement, can also be used with complex media (Figure S13). However, since metabolic effects can be more effectively investigated in defined media, the present study concentrated on the use of defined media.

### 3.4 Influence of different biological systems on DO measurement

In the following experiments, it was tested whether the DO Sensor Pill is also suitable for other biological systems in addition to the measurement of *E*. *coli*. For this purpose, *C. glutamicum*, *K. phaffii*, and *U. maydis* were cultivated. It was further investigated if the biomass measurement could also be integrated here and whether fluorescence measurement could be used as a third parameter.

**FIGURE 6:**
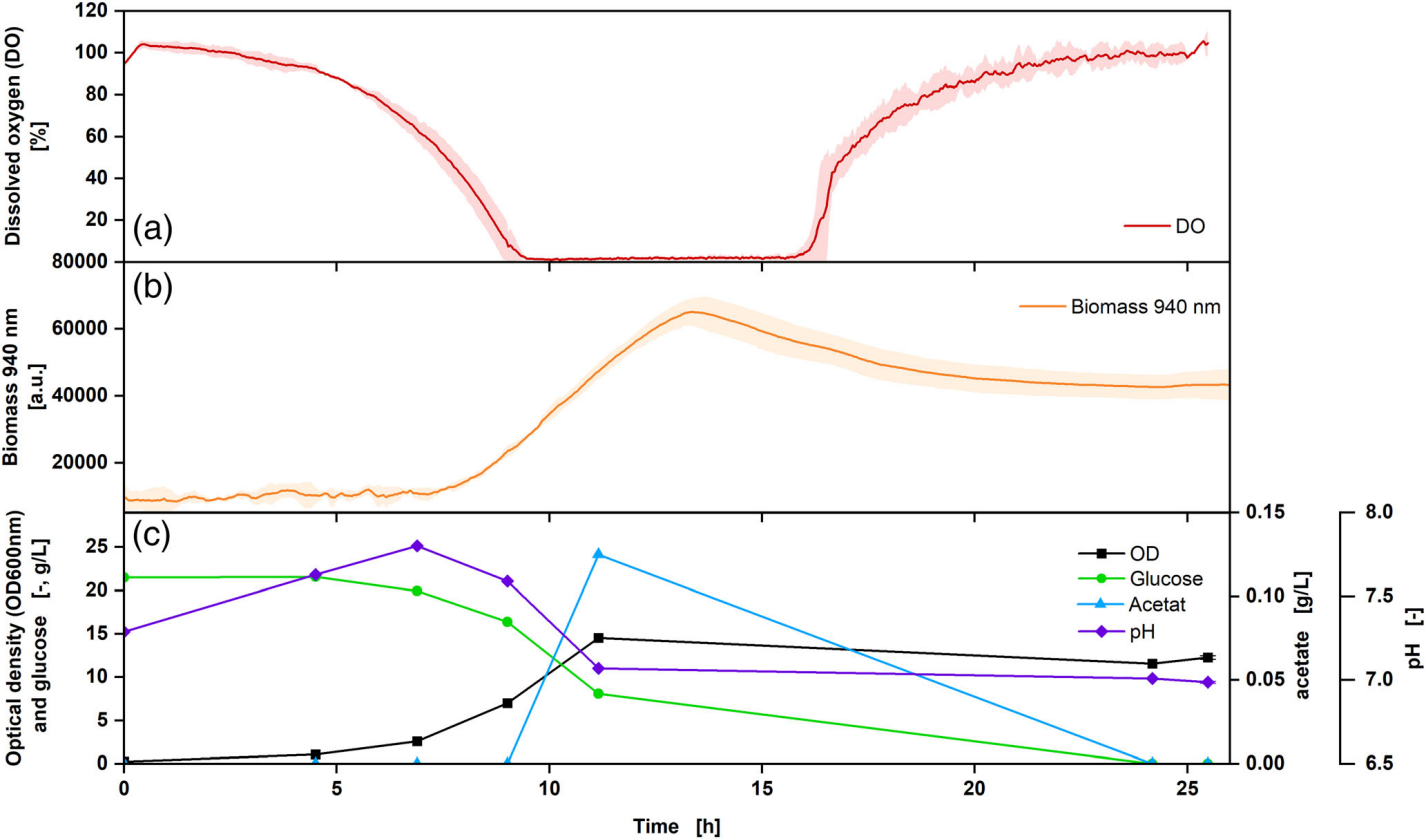
Cultivation of *corynebacterium glutamicum* DM 1933, CG XII medium, DO Sensor Pill (a, red), biomass measurement at 940 nm (b, orange), offline measurement (c): OD_600_ (black), pH (purple), acetate (blue) and glucose (green) concentration, V_L_ = 10%, *n* = 350 rpm, d_0_ = 50 mm, t = 30°C, rep. = 2, OD_600_,_Start_ = 0.2. Shadows indicate the standard deviations.

The results from the *C*. *glutamicum* DM 1933 cultivation can be seen in Figure 6. Oxygen limitation was also present here from approx. 9 h of fermentation and lasts for around 7 h. The biomass measurement shows the typical growth curve of bacterial batch cultivation. The 7 h lag‐phase in the beginning is followed by the exponential growth phase until 12 h, where glucose is depleted. It can also be seen from the HPLC data that acetate was formed during oxygen limitation and was subsequently metabolized again. The stationary growth phase was seen just for a short amount of time (from 12.5 to 13 h). With the following death phase, the biomass concentration decreased, which can be due to cell lysis caused by nutrient depletion or morphological changes, which can influence the backscatter (Figure 6c).^18^, ^45^, ^46^ This can also be observed in the online biomass measurement as the backscatter intensity decreased. Overall, it can be summed up that the online measurements using the DO Sensor Pill and biomass measurement with the MPS correspond well with the offline data (Figure S14).

### 3.5 Influence of intracellular product synthesis on online DO and biomass measurement

**FIGURE 7:**
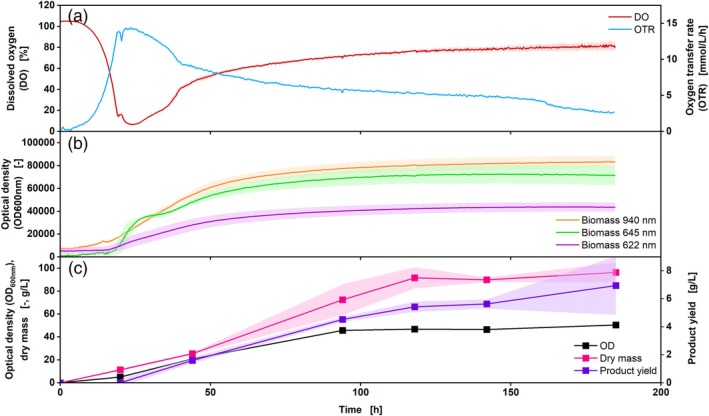
Cultivation of *Ustilago maydis* MB215Δcyp1Δemt1, Verduyn medium for oil production, DO Sensor Pill (a, red), OTR measurement via RAMOS (a, blue), online biomass measurement at 940 nm (b, orange), 645 nm (b, green) and 622 nm (b, light purple), offline measurements: OD_600_ (c, black), dry mass (c, pink), product yield (c, purple). V_L_ = 10%, *n* = 250 rpm, d_0_ = 50 mm, t = 30°C, rep. = 3, OD_600_,_Start_ = 0.2. Shadows indicate the standard deviations.

*U. maydis* was cultivated once in a medium initiating (Figure 7) and once in a medium preventing triglyceride production (Figure S15). Therefore, different glucose and nitrogen concentrations were used (see chapter Material and methods). The influence of intracellular products on online biomass and DO measurement has been investigated. For a better comparison, the OTR results from the RAMOS device are also shown.

Both OTR and DO curves follow the same trend for the first 150 h, including a slower OTR decrease and corresponding a DO increase from 40 to 150 h. The course of the OTR could also be seen by Richter et al., whereas the slower decrease of OTR indicates the phase of triglyceride production.^47^ After 150 h, a further steeper decrease in the OTR was observed, while an increase in DO could not be seen, as not all the glucose had been consumed (Figure S16). When looking at the biomass results in the first 25 h, a steep increase could be measured at 645 nm and 940 nm, which was also seen in the offline measurements. At around 25–34 h, a kink was observed in the online measurement at 645 nm, which could be an indicator of the start of the triglyceride production phase, as this can also be seen in the increase in product yield in the same time range. Additionally, this kink was not observed during cultivation with *U*. *maydis* in medium with sufficient nitrogen supply, which does not trigger triglyceride formation (Figure S15). Furthermore, morphological changes of the cells can also be observed in the backscatter.^18^ As the cells store triglycerides intracellularly, the size of these increases, resulting in a morphological change.^48^ The continuous storage of triglycerides in the cells results in an increase in their size until substrate depletion, a phenomenon that is also evidenced by the online biomass data. An increase in biomass concentration can be ruled out here, as no more growth takes place from the time of nitrogen limitation.^48^ However, this influence should be evaluated positively, as it allows conclusions to be drawn about the process, such as the start of triglyceride production. The onset of triglyceride production cannot be deduced from offline biomass data (OD and dry biomass) as both analyses are affected by intracellular product storage (Figure 7c). During nitrogen limitation, it is assumed that no more biomass growth takes place.^48^ However, this could be surmised from the increase in biomass measured offline. It was shown that this applies to both OD and dry mass measurement. The difference between the increase in biomass and triglyceride concentration can only be determined by analyzing the product, which is time‐consuming and does not provide the data in real‐time.

### 3.6 Influence of GFP production on online DO and biomass measurement and validation of online fluorescence measurement

**FIGURE 8:**
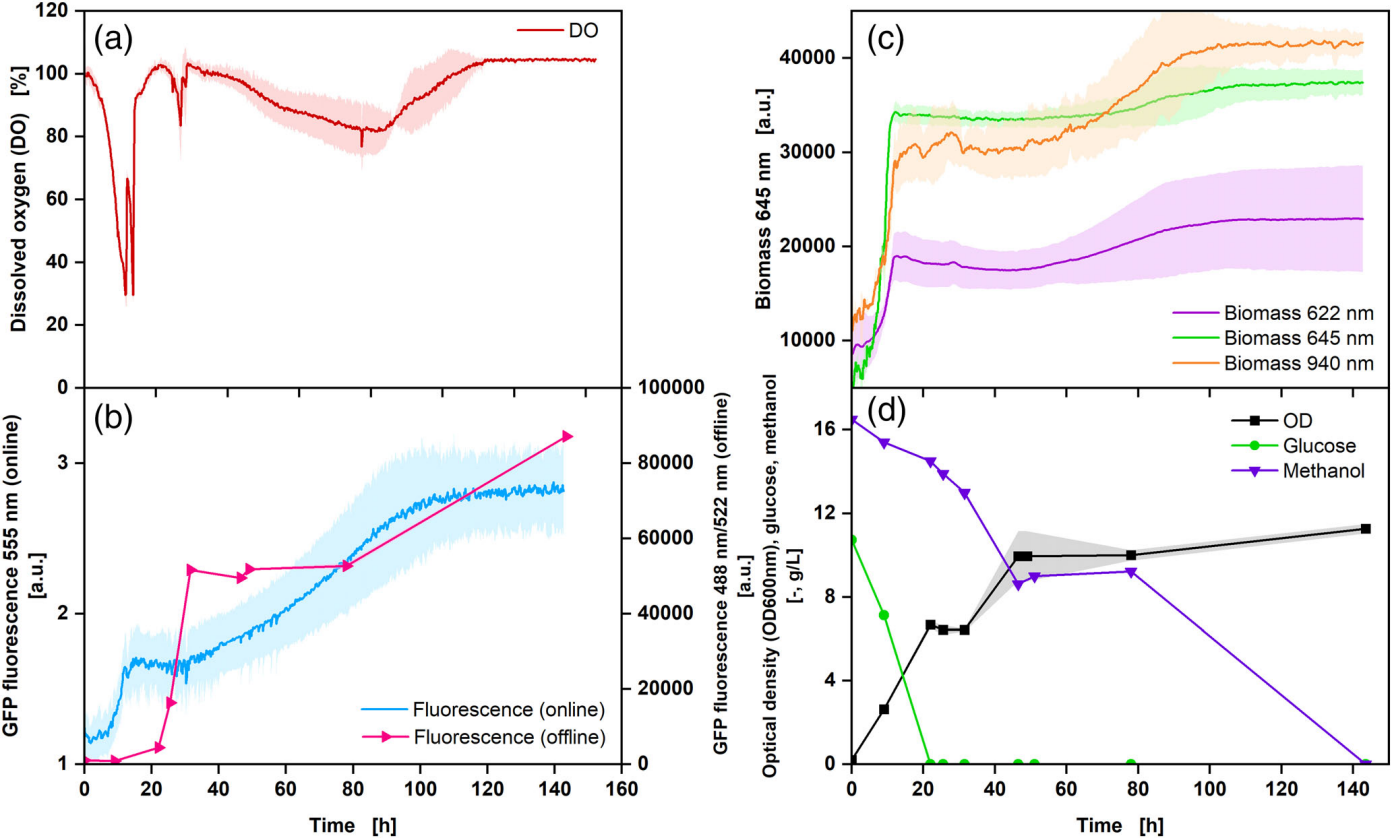
Cultivation of *Komagataella phaffii* Mut^S^, Syn6‐MES medium, DO Sensor Pill (a, red), online fluorescence measurement at 465 nm excitation and 555 nm emission (b, blue) and offline fluorescence measurement at 488 nm excitation and 522 nm emission (b, pink), biomass measurement at 622 nm (c, purple), 645 nm (c, green), 940 nm (c, orange) and offline measurements: OD_600_ (d, black), glucose (d, green), methanol (d, purple) concentration. V_L_ = 10%, *n* = 350 rpm, d_0_ = 50 mm, t = 30°C, rep. = 3, OD_Start_ = 0.2. Shadows indicate the standard deviations.

To test whether the production of GFP protein influences the measurement of DO and/or biomass and if an integration of an online fluorescence measurement is possible, cultivation with the GFP‐producing *K*. *phaffii* Mut^S^ host strain BSYBG11 was performed (Figure 8).

The DO signal shows that no oxygen limitation occurs, but rather two consecutive peaks between 11 and 15 h of cultivation, which can be explained by the Crabtree effect.^49^, ^50^ After approximately 30 h, a further DO peak can be measured, as well as a subsequent slight decrease in DO resulting from the consumption of methanol (Figure 8d).^50^ The DO increases at the end, as methanol is depleted. For the online fluorescence measurement, emission wavelengths of 515, 555, and 590 nm were selected (only 555 nm is shown, further data: Figure S17). The excitation wavelength was set to 465 nm. In parallel, samples were taken for the offline determination of fluorescence. An increase in emission measured in the offline samples can be observed at the beginning of cultivation from 20 h on. GFP was already formed here, which is also reflected by the decrease in the methanol concentration, resulting from a GFP production due to the basal expression described by Wollborn et al.^50^ The second increase of the online measured emission aligns well with the main GFP production phase from 30 h onwards. A discrepancy between online and offline fluorescence measurements could be observed. While the fluorescence measured online increased from 5 to 14 h of cultivation, an increase was only measured in the offline biomass measurement after 22 h of cultivation. The impact of the fluorescence measurement by the online biomass measurement via backscatter can be disregarded, as the measurements are taken sequentially. This is also taken into consideration when setting the measurement intervals in the software. In this instance, the discrepancy between online and offline fluorescence measurements can be attributed to the bandwidth of the measurements. It is possible to set different detection spectra for both online and offline measurements, with the desired peak detection wavelength being selected. However, a bandwidth is always measured in addition to this peak, with a size that depends on the spectrometer or detector. In this instance, the bandwidth for the spectrometer in the MPS at a wavelength of 555 nm was found to be ±27 nm, whereas the bandwidth for the plate reader used for offline samples was only ±2 nm.^28^ This suggests the potential for the detection of additional fluorophores during the online measurement, resulting in a discrepancy. Furthermore, biological replicates were conducted during the online measurement, while technical replicates were performed during the offline measurement. Particularly, the offline measurement of the sampling point after 31.5 h stands out, which may be attributed to technical replicates. In general, a less detrended course can be demonstrated by a lower number of sampling points. This once more demonstrates the significance of continuous online measurements during biological processes in the shake flask. The OD course demonstrates an increase from 0 to 15 h of cultivation, which can also be seen in the online biomass data at all wavelengths (Figure 8c,d). However, larger deviations between the triplicates can be measured at 940 and 622 nm. Furthermore, the backscatter intensity increases significantly after 75 h of cultivation, where only a slight increase in offline OD values was measured. The glucose is also already consumed after 20 h, indicating that no additional biomass is measured at these wavelengths. The observed change could be attributed to the presence of metabolites and alterations in cell conformation, which occur during this phase of cultivation and influence the backscatter within these wavelength ranges. A slight increase in OD, which was also measured at 645 nm, might be caused by the incorporation of methanol into peroxisomes and additional growth on methanol.^51^ Consequently, the measurement at 645 nm provides the most significant values. An additional assumption is that there is a crosstalk between the excitation, emission peaks, and the backscatter from the fermentation broth in the first 20 h of fermentation, as here the biomass increases. The three DO measurement replicates show greater deviation during the main phase of GFP production, likely due to methanol evaporation. Variations in flask tightness could lead to differing methanol loss, affecting DO measurements, as methanol acts as an inducer for GFP and stimulates cellular respiratory activity. The possibility to simultaneously measure DO, biomass, and fluorescence in one flask/system provides further insights into the bioprocess. In addition, different wavelengths can be set for excitation and emission, as well as for biomass measurement, and recorded in parallel, which further increases the range of applications and could avoid interferences of measurements. However, for unknown systems, screening should be carried out regarding the appropriate wavelength range for biomass measurement (backscatter) and fluorescence measurement (excitation/emission) to obtain the optimal settings. A simultaneous measurement of dissolved oxygen (DO), biomass, and fluorescence can also be conducted in complex media (Figure S13). However, due to the challenges in identifying metabolic effects from online data, this aspect was not included in this study.

## 4 CONCLUSION

Shake flasks are the most commonly used vessel for biotechnological research and process development. For this reason, it is urgent to better understand shake flask cultivations, which is possible through online monitoring of parameters like dissolved oxygen, oxygen transfer rate, and biomass. In recent years, the development of technologies for online measurement has increased. However, only single parameters such as oxygen solubility or the oxygen transfer rate are usually recorded. In this work, it was determined whether it is possible to gain more crucial insights into the bioprocess by analyzing dissolved oxygen, biomass, and fluorescence online and simultaneously in parallel in a single shake flask with the Multiparameter Sensor (MPS). Abiotic tests showed good reproducibility, as well as sensitivity and accuracy to changing conditions concerning oxygen solubility, measured with the DO Sensor Pill. This was also the case in all biological systems. In comparison with existing measuring systems for determining oxygen solubility and oxygen transfer rate, the DO Sensor Pill showed the same results. Reproducibility was demonstrated for online DO, biomass, and fluorescence measurement throughout all experiments. The observed discrepancies between online and offline fluorescence measurements may be attributed to differences in the bandwidths of the spectrometers integrated into the MPS and the plate reader. In conclusion, this study shows that it is possible to measure three different parameters online in parallel with the easy‐to‐use MPS for a wide range of biological systems, gaining detailed insights into the process. Shake flasks have well‐known benefits like a high degree of parallelization, sufficient volume for offline analysis, easy handling, and low cost per experiment. Combining these advantages with the advanced insights gained from the MPS has the potential to further strengthen the shake flask's position in crucial applications such as bioprocess characterization during process development and scale‐up.

## AUTHOR CONTRIBUTIONS

**Lara Strehl:** Designed the study, performed the experiments, analyzed the data, and drafted the manuscript. **Anna‐Lena Kuhn:** Designed the study, performed the experiments, analyzed the data, and drafted the manuscript. **Kyra Hoffmann:** contributed to the conception, reviewed and edited the manuscript, and provided the equipment. **Jørgen Barsett Magnus and Marcel Mann:** supervised and initiated the study, participated in data interpretation, and assisted in drafting the manuscript. All authors read and approved the final manuscript.

## FUNDING INFORMATION

aquila biolabs GmbH, Arnold‐Sommerfeld‐Ring 2, 52499 Baesweiler—Germany.

## CONFLICT OF INTEREST STATEMENT

The authors declare the following financial interests/personal relationships that may be considered potential competing interests: Lara Strehl reports equipment, drugs, or supplies were provided by aquila biolabs GmbH, Arnold‐Sommerfeld‐Ring 2, 52,499 Baesweiler, Germany. Anna‐Lena Kuhn reports equipment, drugs, or supplies were provided by aquila biolabs GmbH, Arnold‐Sommerfeld‐Ring 2, 52,499 Baesweiler, Germany. Kyra Hoffmann reports a relationship with aquila biolabs GmbH, Arnold‐Sommerfeld‐Ring 2, 52,499 Baesweiler, Germany that includesemployment. If there are other authors, they declare that they have no known competing financial interests or personal relationships that could have appeared to influence the work reported in this paper.

## Supporting information


**Data S1.** Supporting Information.

## Data Availability

The data that support the findings of this study are available from the corresponding author upon reasonable request.
