# Supplementary material for: A novel multiparameter sensor for shake flask cultivations: Online biomass, dissolved oxygen, and fluorescence monitoring for comprehensive bioprocess characterization
Source: Biotechnol Prog. 2025 Apr 23;41(5):e70035. doi: 10.1002/btpr.70035 (PMC12531943; doi:10.1002/btpr.70035)
Supplement: Supplementary file 1 — Data S1. Supporting Information. [file BTPR-41-e70035-s001.docx]

**Supplementary Material**

**
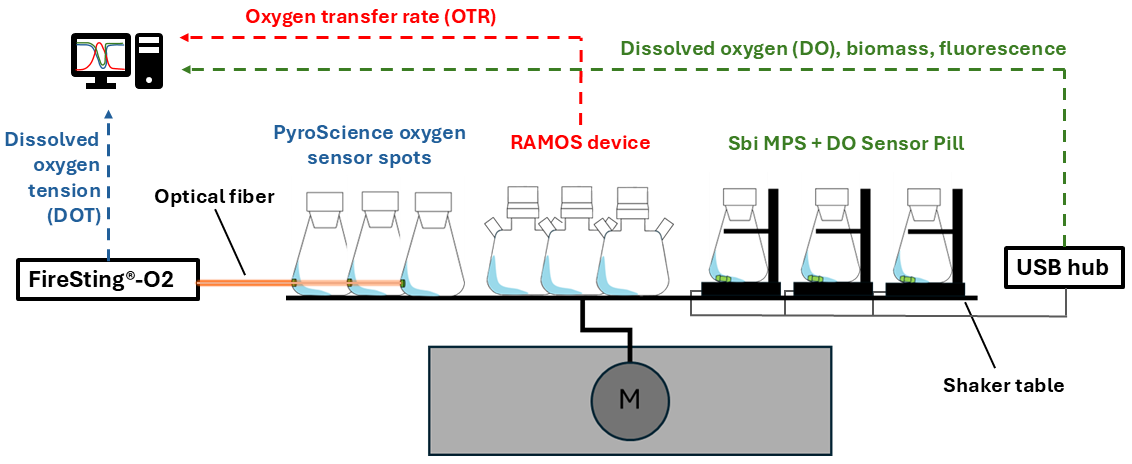
**

Figure S 1 Experimental setup for simultaneous measurements of dissolved oxygen (DO) by oxygen sensor spots (PyroScience) and DO Sensor Pill + MPS (sbi) and oxygen transfer rate (OTR) by RAMOS device (inhouse build) (offline shake flasks for the sake of clarity not shown)

Table S 1 Further information on the DO Sensor Pill

| **Further information on the DO Sensor Pill** |
| --- |
| Single-use product |
| Calibration by the manufacturer |
| Sterilized by the manufacturer |
| Shelf life: 6 months in original packaging |

| A | 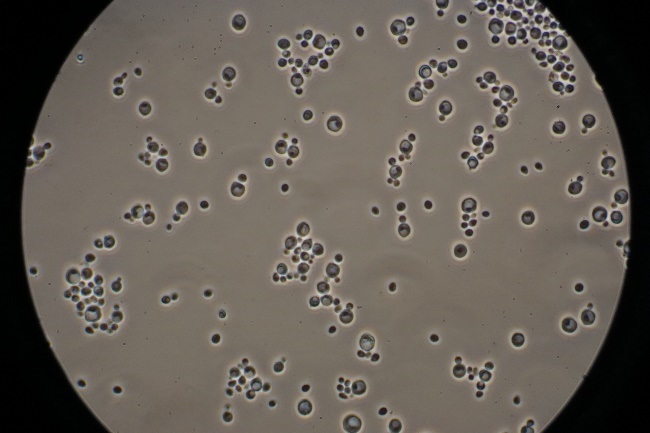 | 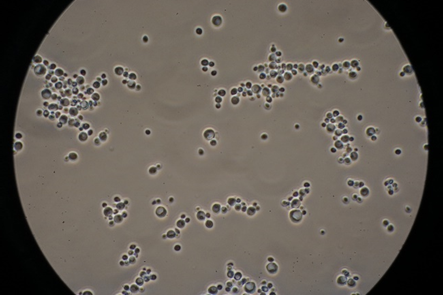 |
| --- | --- | --- |
| B | 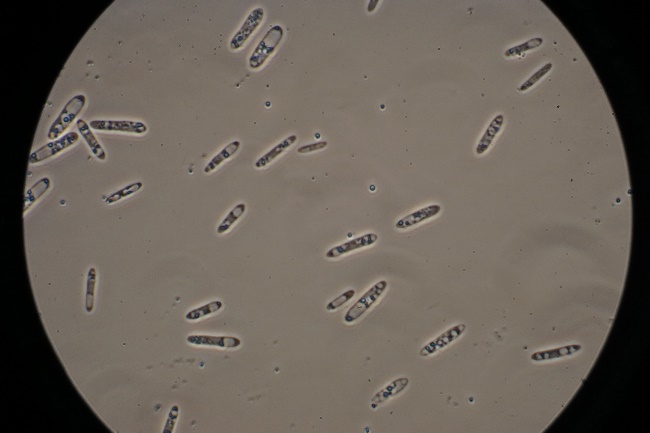 | 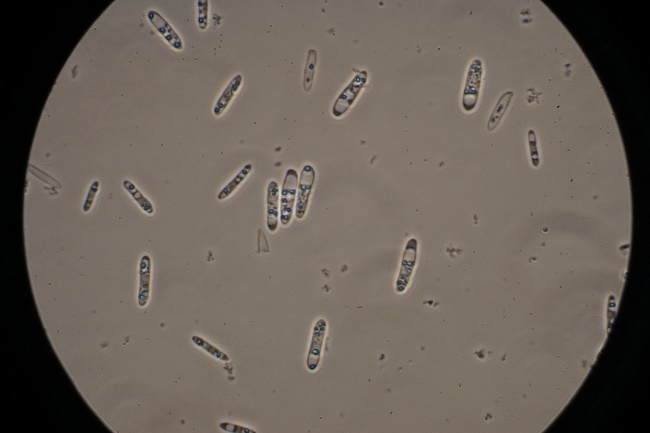 |

Figure S 2 Microscopic picture of A: Komatagella phaffii after incubation without the DO Sensor Pill (left) and after incubation with the DO Sensor Pill (right), B: Ustilago maydis after incubation without the DO Sensor Pill (left) and after incubation with the DO Sensor Pill (right).

OTR calculated from DOT measurements

The DO is calculated from the OTR with the equation Eq 1 [3]:

$$DOT= \left( \frac{p_{O_{2}}^{gas}}{p_{O_{2}}^{cal}}-\left( \frac{OTR}{k_{L}a*\frac{L_{O_{2}}}{p_{O_{2}}^{cal}}} \right) \right)*100$$

Eq 1

Table S 2 Medium composition of Wilms-MOPS media for cultivation of Escherichia coli. Solutions were stored at room temperature if not stated otherwise. The solutions were sterilized through autoclavation or by sterile filtration through a 0.2 µm cut-off cellulose acetate membrane filter. If not stated otherwise, all media components were dissolved in DI water

| **Component** | **Final concentration [g/L] / [mL/L]** |  |
| --- | --- | --- |
| (N-morpholino)-propane sulfonic acid (MOPS) | 41.85 |  |
| (NH_4_)_2_SO_4_ | 6.98 |  |
| H_2_HPO_4_ | 3 |  |
| Na_2_SO_4_ | 2 |  |
| Glucose | 20 (preculture) 10 (main culture) |  |
| Thiamin solution | 1 |  |
| MgSO_4_-solution | 10 |  |
| Trace element solution | 1 |  |
| **Thiamin solution** (stored at 4 °C) | | |
| Thiamine chloride | 10 |  |
| **MgSO_4_-solution** | | |
| MgSO_4_ | 50 |  |
| **Trace element solution** (stored at 4 °C) | | |
| FeCl_3_*6H_2_O | 41.76 |  |
| Na_2_EDTA (Triplex III) | 33.4 |  |
| CaCl_2_*2H_2_O | 1.98 |  |
| ZnSO_4_*7H_2_O | 0.54 |  |
| CoCl_2_*6H_2_O | 0.54 |  |
| CuSO_4_*5H_2_O | 0.48 |  |
| MnSO_4_*H_2_O | 0.3 |  |

Table S 3 Medium composition of the YPG complex medium for pre-cultivation and the modified CG-XII medium for main cultivation of Corynebacterium glutamicum. Solutions were stored at room temperature if not stated otherwise. The solutions were sterilized through autoclavation or by sterile filtration through a 0.2 µm cut-off cellulose acetate membrane filter. If not stated otherwise, all media components were dissolved in DI water

| **Component** | **Final concentration [g/L] / [mL/L]** |
| --- | --- |
| **Complex pre-cultivation medium (YPG)** | |
| Yeast extract | 10 |
| Peptone | 10 |
| NaCl | 2.5 |
| MgSO4*7H2O | 0.25 |
| Glucose | 20 |
| **CG-XII medium** | |
| Glucose | 20 |
| (NH_4_)_2_SO_4_ | 10 |
| KH_2_PO_4_ | 2 |
| K_2_HPO_4_ | 1 |
| MgSO_4_*7 H_2_O | 0.25 |
| MOPS buffer | 21 |
| CO(NH_2_)_2_ | 2 |
| CaCl_2_ H_2_O | 0.01 |
| Biotin solution | 0.0002 |
| d-biotin diluted in 50 (v/v)% 2-propanol and DI water, stored at -20 °C | |
| Trace element solution | 1 |
| 3,4-dihydroxybenzoic acid | 0.03 |
| 3,4-dihydroxygenzoic acid diluted in 10 (w/v) % of NaOH and DI water, stored at -20 °C | |
| **Trace element solution** (stored at 4 °C) | |
| FeSO_4_*7 H_2_O | 10 |
| MnSO_4_*H_2_O | 10 |
| ZnSO_4_*7 H_2_O | 1 |
| CuSO_4_ | 0.02 |
| NiCl_2_*6H_2_O | 0.002 |

Table S 4 Medium composition of Syn6-MES medium for cultivation of Komatagella phaffii. Solutions were stored at room temperature if not stated otherwise. The solutions were sterilized through autoclavation or by sterile filtration through a 0.2 µm cut-off cellulose acetate membrane filter. If not stated otherwise, all media components were dissolved in DI water

| **Component** | **Amount]** |
| --- | --- |
| **Basic medium** | **Concentration [g/L]** |
| 2-(N-morpholino)-ethanesulfonic acid (MES) | 91 |
| KCl | 11 |
| MgSO_4_*7 H_2_O | 10 |
| KH_2_PO_4_ | 3,33 |
| NaCl | 1,1 |
| **Glucose solution** | |
| Glucose | 500 |
| **(NH_4_)_2_SO_4_ solution** | |
| (NH_4_)_2_SO_4_ | 383 |
| **CaCl_2_ solution** | |
| CaCl_2_ | 100 |
| **Microelement solution** | |
| Na_2_EDTA Titriplex III | 6.65 |
| (NH_4_)_2_Fe(SO_4_)_2_ | 6.65 |
| MnSO_4_*H_2_O | 2.65 |
| ZnSO_4_*7H_2_O | 2.0 |
| CuSO_4_*5 H_2_O | 0.55 |
| **Vitamin solution** (stored at 4 °C) | |
| Thiamine chloride | 148.33 |
| d-biotin | 4 |
| d-biotin was dissolved in 10 mL of a mixture of a (1:1) 2-propanol and DI-water | |
| **Trace element solution** (stored at 4 °C) | |
| NiSO_4_*6H_2_O | 0.065 |
| CoCl_2_*6H_2_O | 0.065 |
| Boric acid | 0.065 |
| KI | 0.065 |
| Na_2_MoO_4_*2H_2_O | 0.065 |

Table S 5 Medium composition of the modified Verduyn medium for cultivation of Ustilago maydis. Pre-culture with 20 g/L glucose, main culture with 100 g/L glucose. Solutions were stored at room temperature if not stated otherwise. The solutions were sterilized through autoclavation or by sterile filtration through a 0.2 µm cut-off cellulose acetate membrane filter. If not stated otherwise, all media components were dissolved in DI water

| **Component** | **Concentration [g/L]** |  |
| --- | --- | --- |
| (NH_4_)_2_SO_4_ | 5 (pre-culture) or 1.6 (main culture) |  |
| MgSO_4_ | 0.5 |  |
| FeCl_3_*6H_2_O | 0.01 |  |
| Glucose | 20 (preculture) or 100 (main culture) |  |
| 2-(N-morpholino)-ethanesulfonic acid (MES) | 0.4 molarity |  |
| Trace element solution | 1 |  |
| **Trace element solution** (stored at 4 °C) | | |
| EDTA | 15 |  |
| FeSO_4_*H_2_O | 3 |  |
| MnCl_2_*2H_2_O | 0.84 |  |
| ZnSO_4_*7H_2_O | 4.5 |  |
| CuSO_4_*5H_2_O | 0.3 |  |
| CoCl_2_*6H_2_O | 0.3 |  |
| Na_2_MoO_4_*2H_2_O | 0.4 |  |
| CaCl_2_*2H_2_O | 4.5 |  |
| H_3_BO_3_ | 1. |  |
| KI | 0.1 |  |


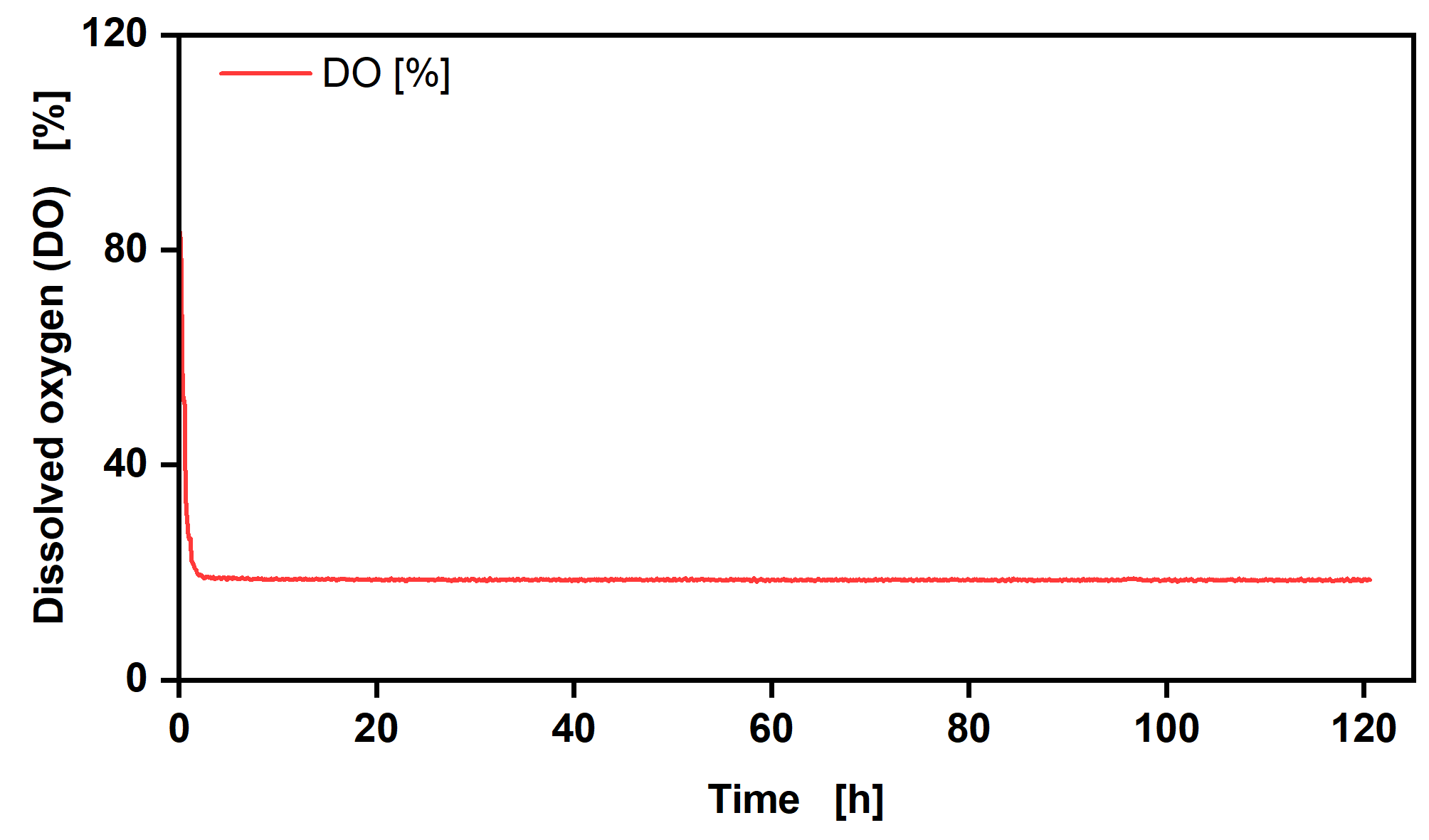


Figure S 3 Measured dissolved oxygen (DO) concentration in an abiotic Wilms-MOPS medium in RAMOS shake flasks, gassed with a gas trap with a gas composition of: 1.3% CO_2_, 3.4% O_2_, 5.9% CH_4_ and 89.4% N_2_. Calculation of DO measured by the DO Sensor Pill (calibrated at 20.95% oxygen concentration): 20.95%/ 100 % ·18% = 3.4% O_2, calculated_. Cultivation conditions: n: 250 rpm, V_L_: 10%, d_0_: 50 mm, t: 30 °C.


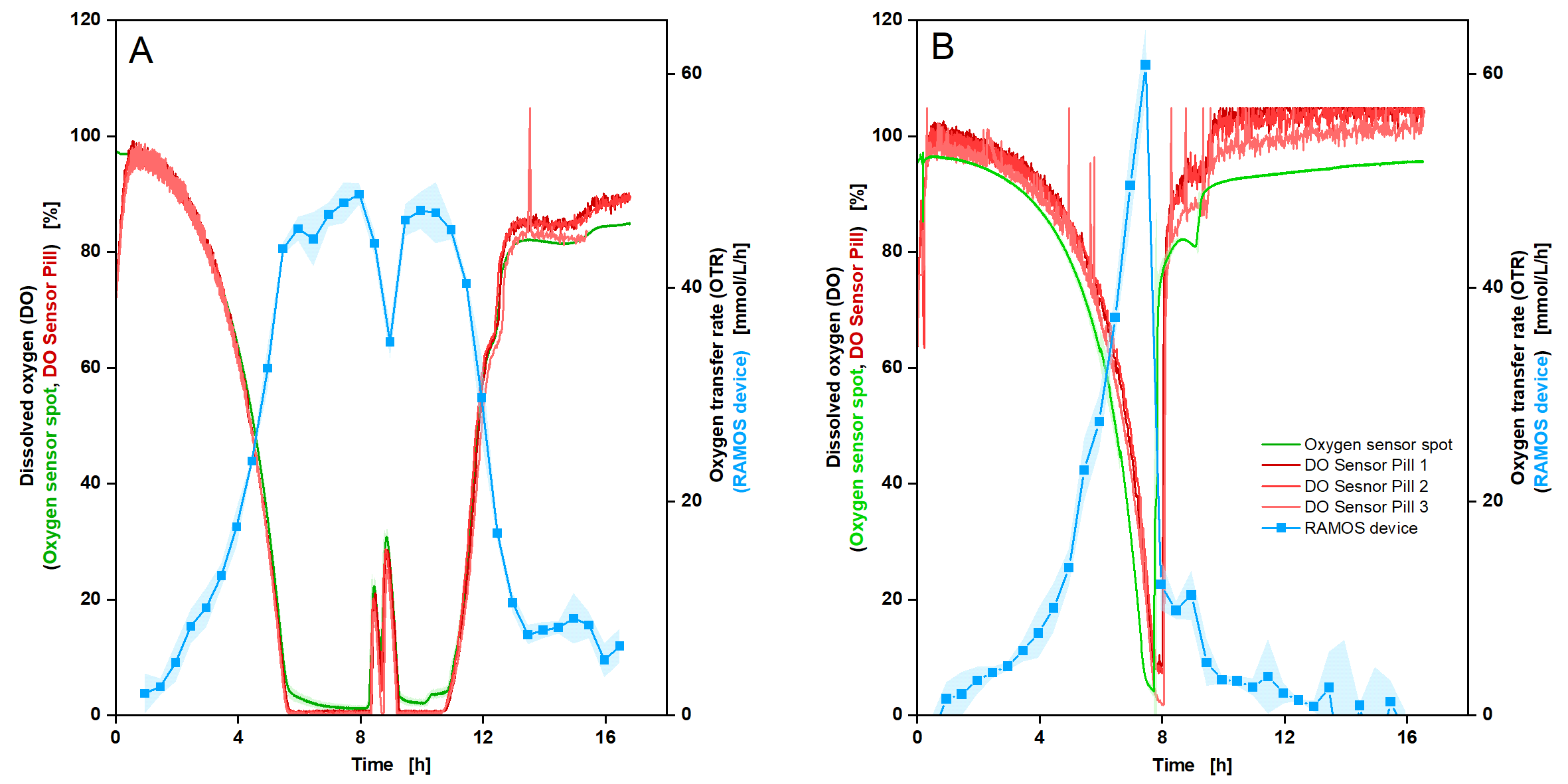


Figure S 4 Comparison of dissolved oxygen (DO) and oxygen transfer rate (OTR) measurements from the measuring systems: DO Sensor Pill, oxygen sensor spot, and RAMOS device with Escherichia coli BL 21 DE 3. A: Wilms-MOPS medium, V_L_ = 10%, n = 250 rpm, d_0_ = 25 mm, t = 37 °C, rep._Pill_ = 3, rep._PyroScience_ = 2, rep._RAMOS_= 2, OD_600,Start_ = 0.1. B: Wilms-MOPS medium, V_L_= 4%, n = 350 rpm, d_0_ = 25 mm, t = 37 °C, rep._Pill_ = 3, rep._PyroScience_ = 2, rep._RAMOS_ = 2, OD_600,Start_ = 0.1.


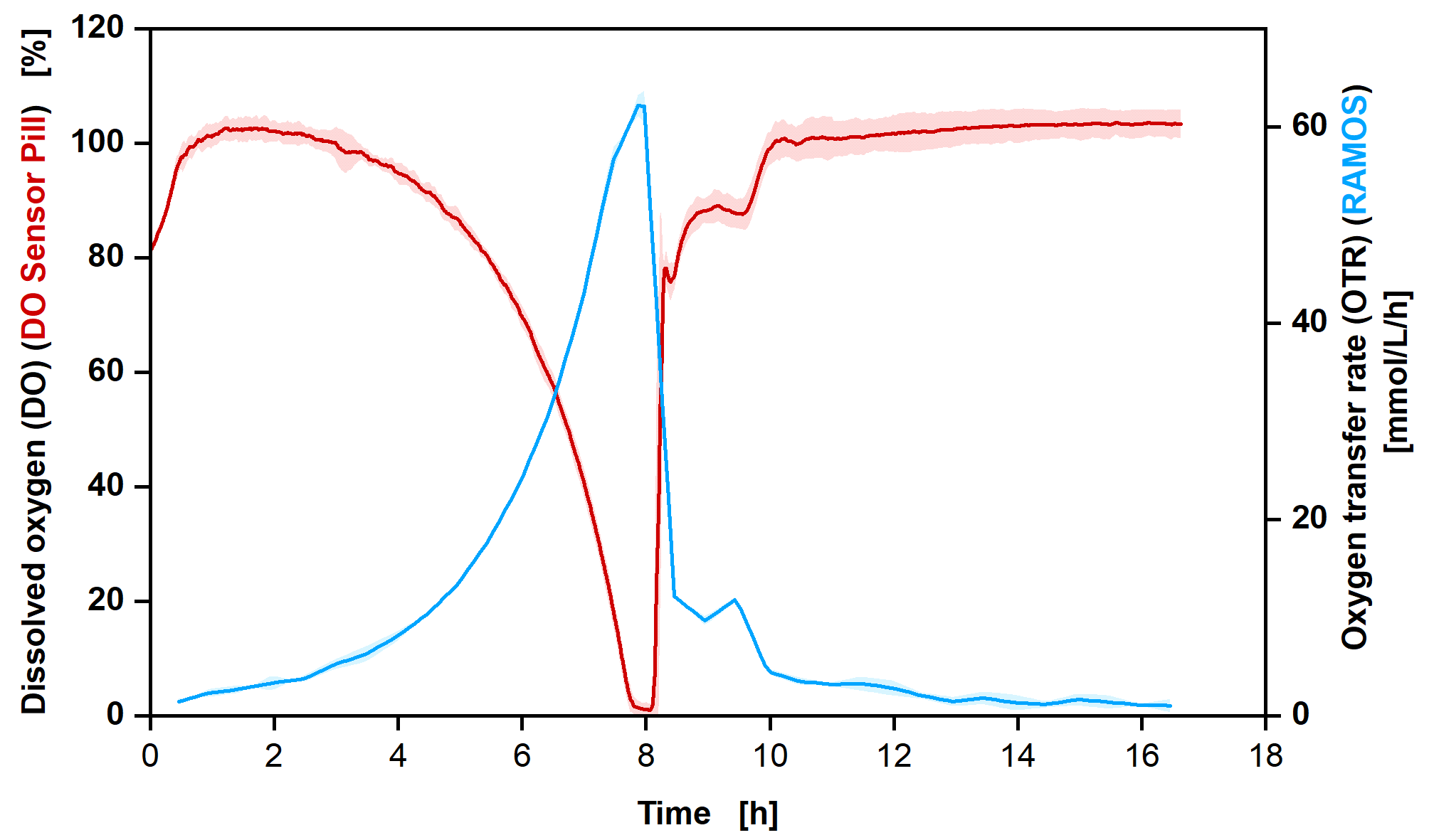


Figure S 5 Comparison of dissolved oxygen (DO) and oxygen transfer rate (OTR) measurements from the measuring systems: DO Sensor Pill and RAMOS device with Escherichia coli BL 21 DE 3. Wilms-MOPS medium, V_L_= 4%, n = 350 rpm, d_0_ = 25 mm, t = 37 °C, rep._Pill_ = 3, rep._RAMOS_ = 2, OD_600,Start_ = 0.1.


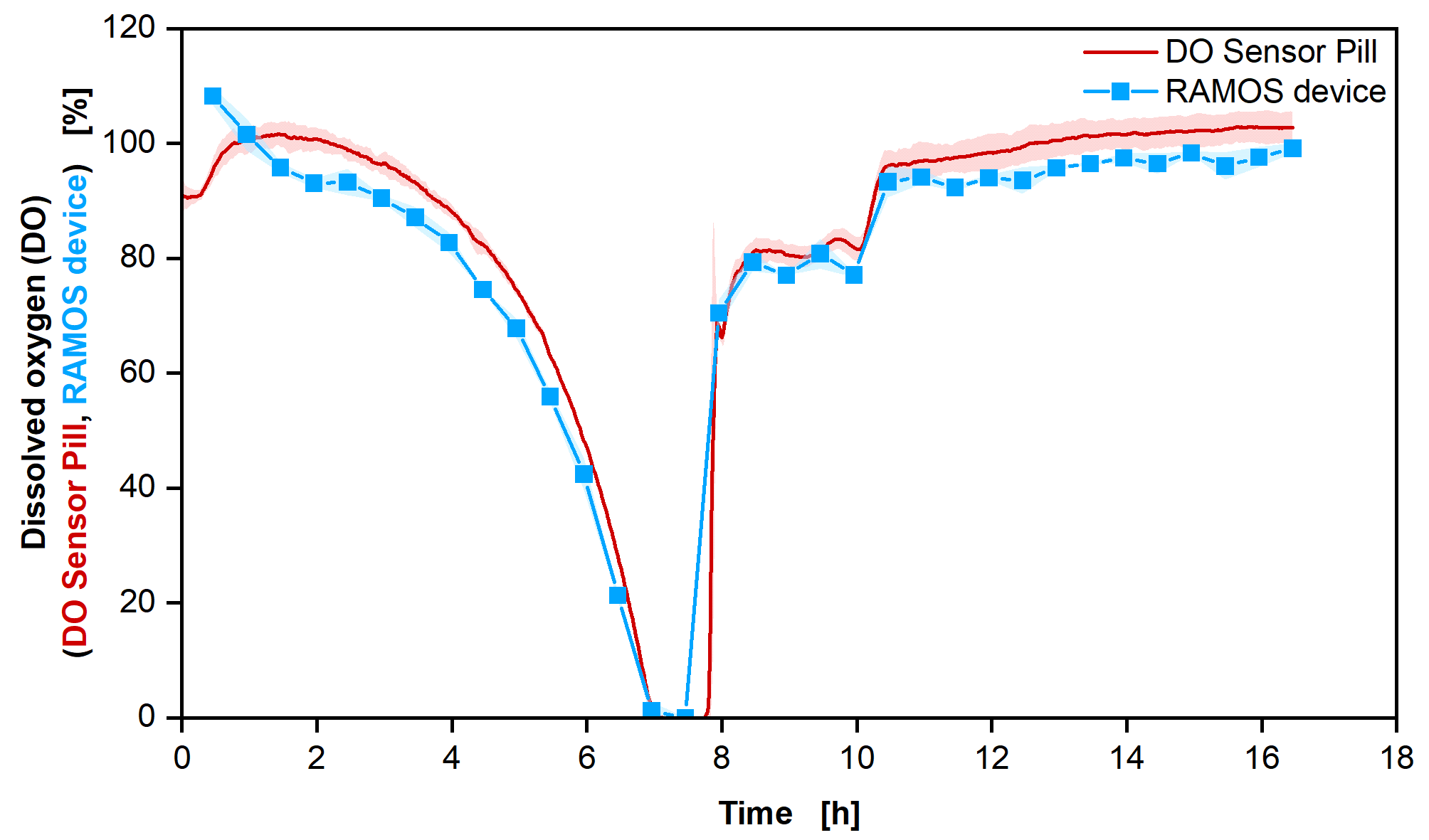


Figure S 6 Comparison of dissolved oxygen (DO) and oxygen transfer rate (OTR) measurements from the measuring systems: DO Sensor Pill and RAMOS device with Escherichia coli BL 21 DE 3. Wilms-MOPS medium, V_L_= 5%, n = 350 rpm, d_0_ = 50 mm, t = 37 °C, rep._Pill_ = 3, rep._RAMOS_ = 2, OD_600,Start_ = 0.1.


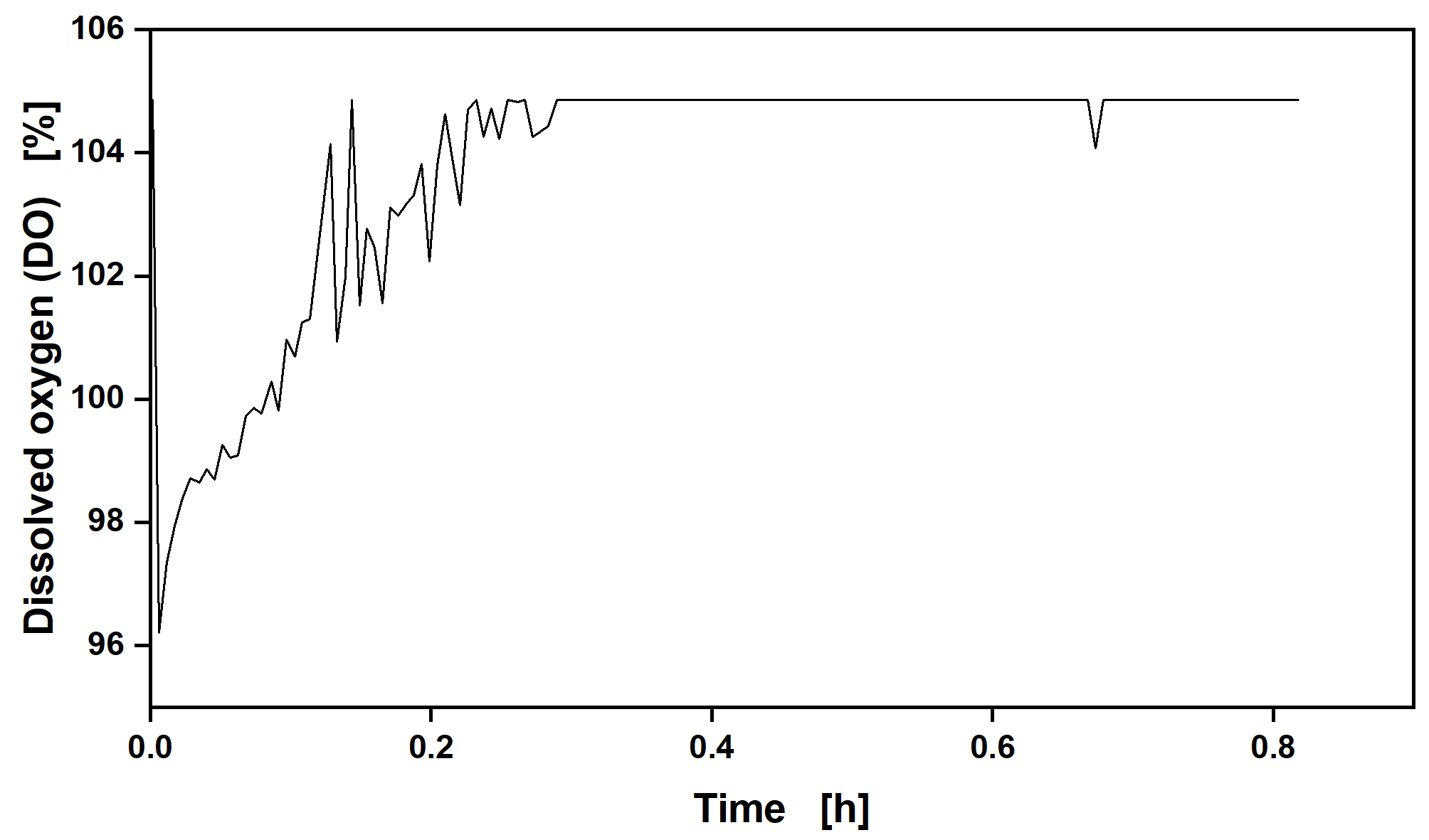


Figure S 7 Detailed view on the DO measured by the DO Sensor Pill directly after the start of the incubation. Abiotic system in Wilms-MOPS medium. Cultivation conditions: n: 250 rpm, V_L_: 10%, d_0_: 50 mm, t: 30 °C.


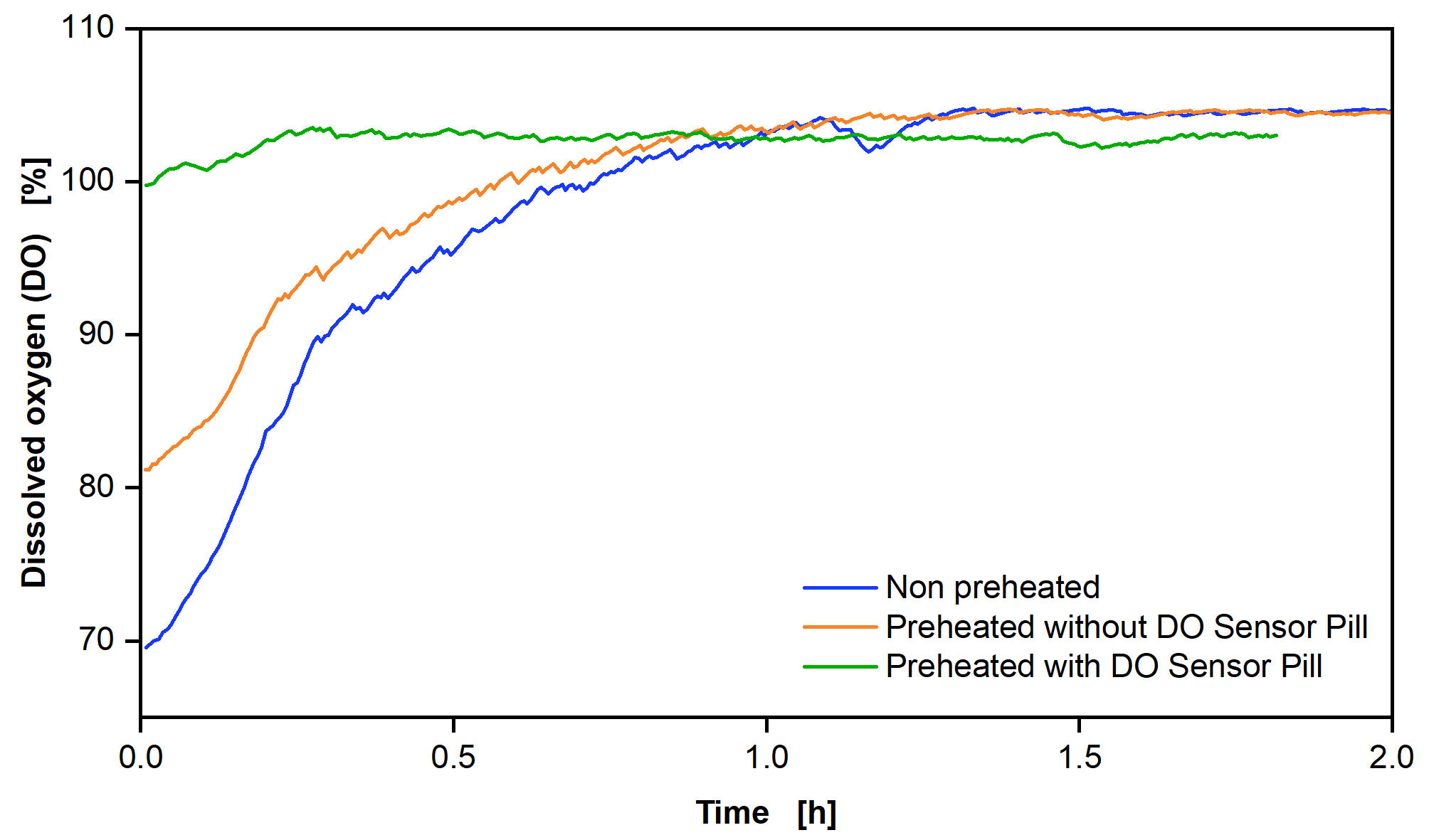


Figure S 8 Adaptation test of the DO Sensor Pill. Abiotic system in dist. water. Non-preheated: flask was filled with water and DO Sensor Pill and placed directly in the shaker and data recording was started, Preheated without DO Sensor Pill: flask with water was placed in the shaker until the desired temperature was reached, then the DO Sensor Pill was added and data recording was started, Preheated with DO Sensor Pill: flask with water and DO Sensor Pill was placed in the shaker until the desired temperature was reached, then data recording was started. Cultivation conditions: n: 250 rpm, V_L_: 10%, d_0_: 50 mm, t: 37 °C.


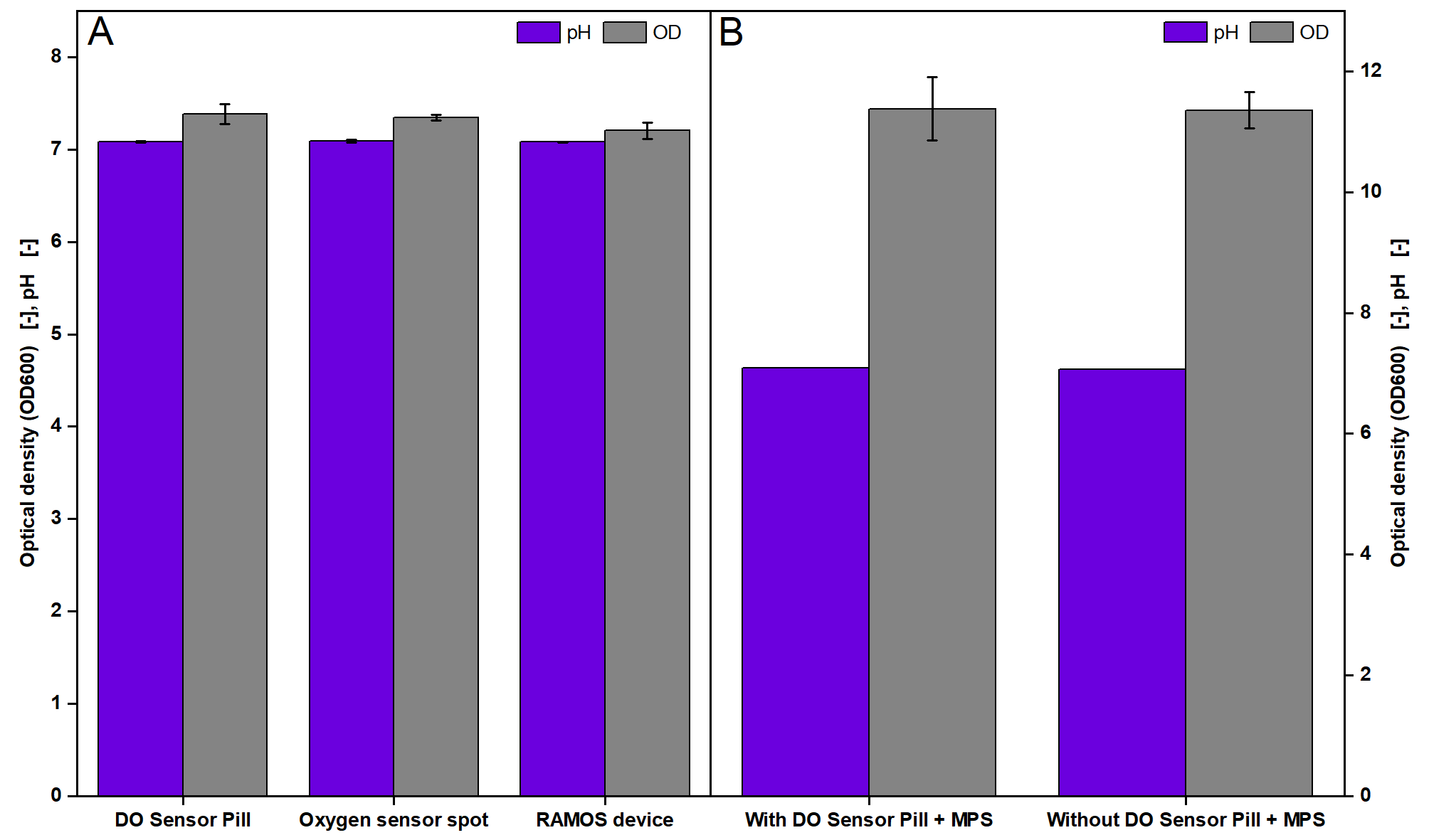


Figure S 9 Comparison of OD_600_ (grey) and pH (purple) between the cultivations of E. coli BL 21 DE 3, A: with the DO sensor pill, the oxygen sensor spots, and the RAMOS device, and B: with and without the DO sensor pill and the MPS. Cultivation conditions can be found in figure 3 A and B in the main manuscript.


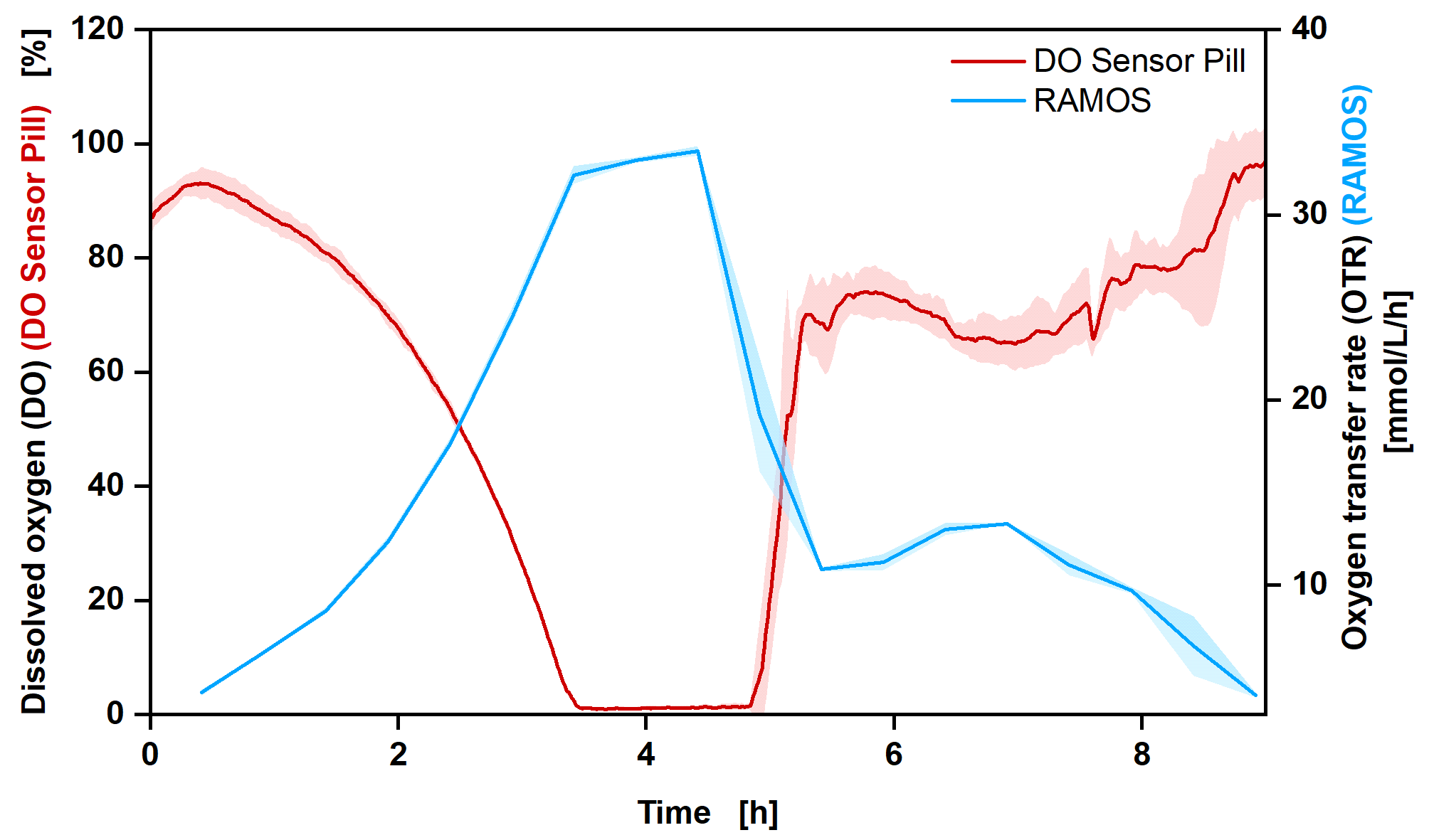


Figure S 10 Cultivation of Escherichia coli BL 21 DE 3, Wilms MOPS medium, DO Sensor Pill (red) and RAMOS (blue), V_L_ = 10%, n = 350 rpm, d_0_ = 50 mm, t = 37 °C, rep. = 3, OD_600,Start_= 0.5. Shadows indicate the standard deviations (Figure 4, main manuscript)


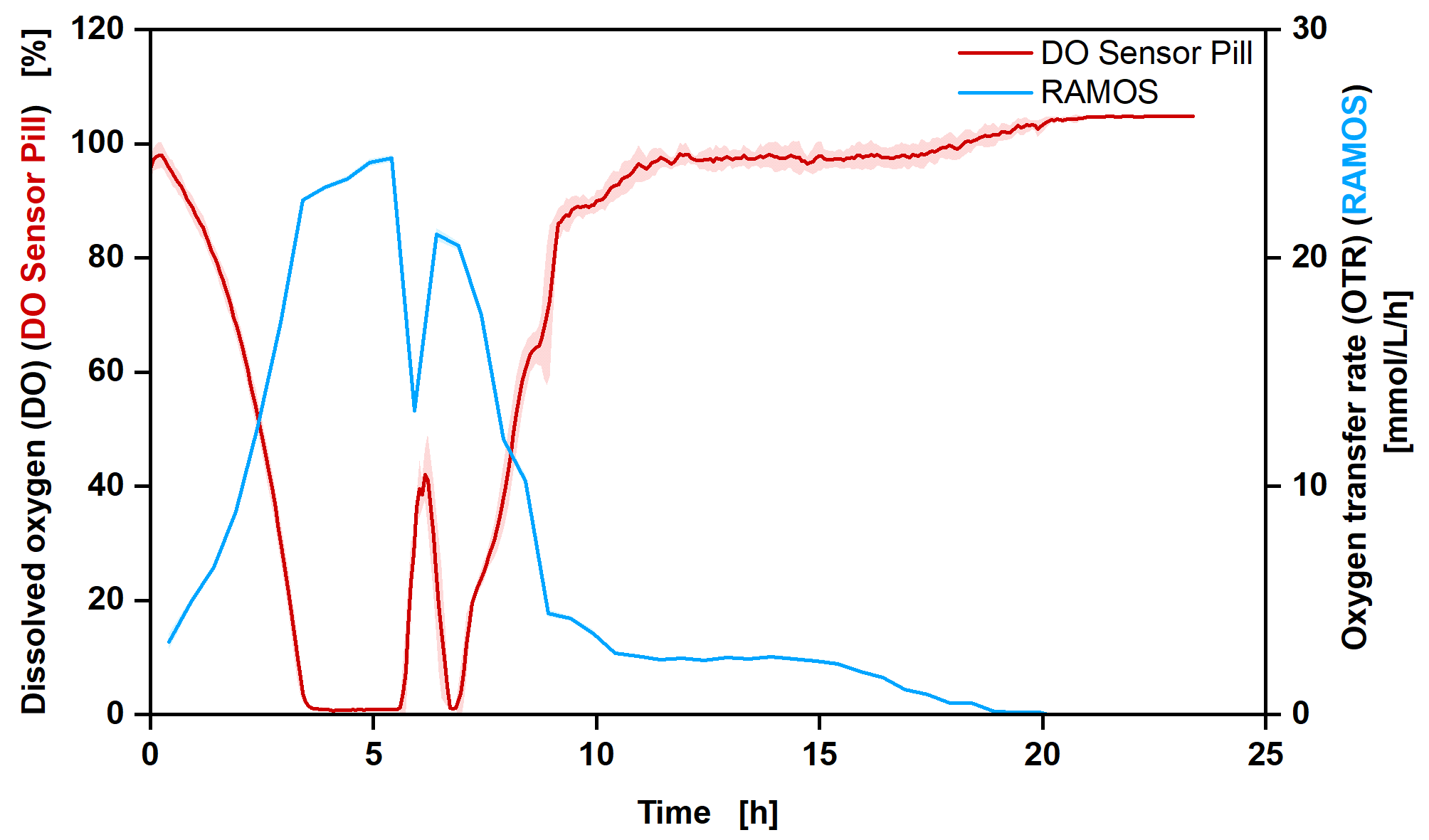


Figure S 11 Cultivation of Escherichia coli BL 21 DE 3, Wilms MOPS medium, DO Sensor Pill (red) and RAMOS (blue), V_L_ = 10%, n = 250 rpm, d_0_ = 50 mm, t = 37 °C, rep. = 3, OD_600,Start_= 0.5. Shadows indicate the standard deviations (Figure 5, main manuscript)


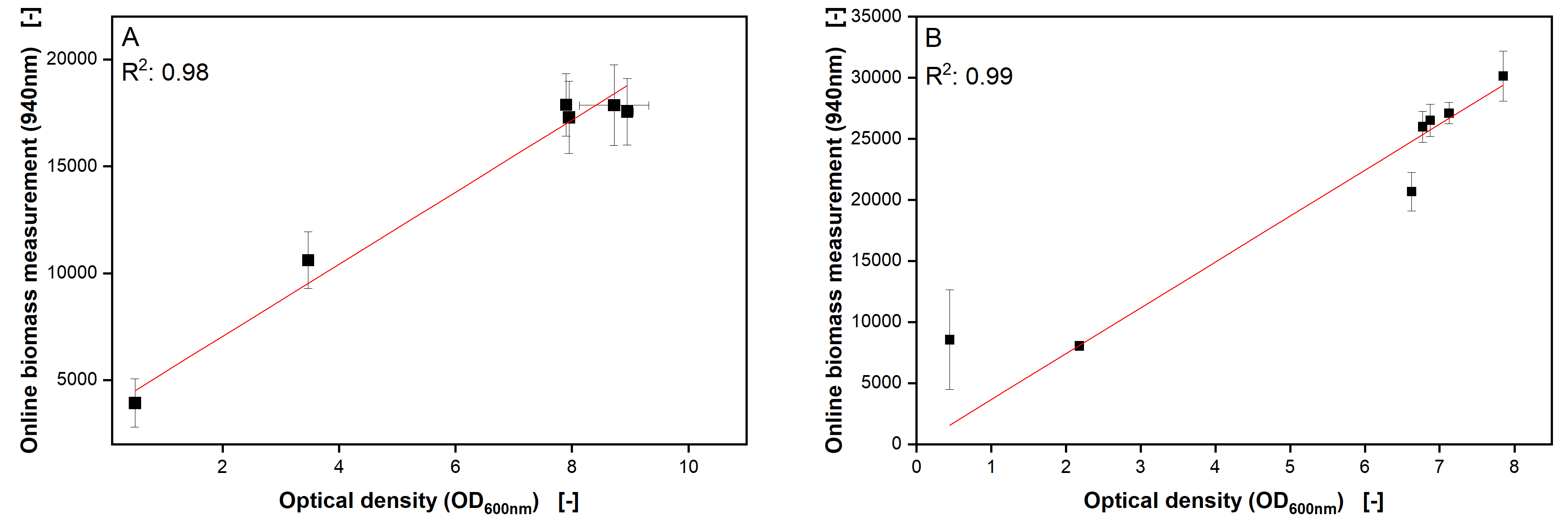


Figure S 12 Correlation between online measured backscatter at 940 nm and offline measured OD at 600 nm, A: cultivation at 350 rpm (Figure 4, main manuscript), B: cultivation at 250 rpm (Figure 5, main manuscript)


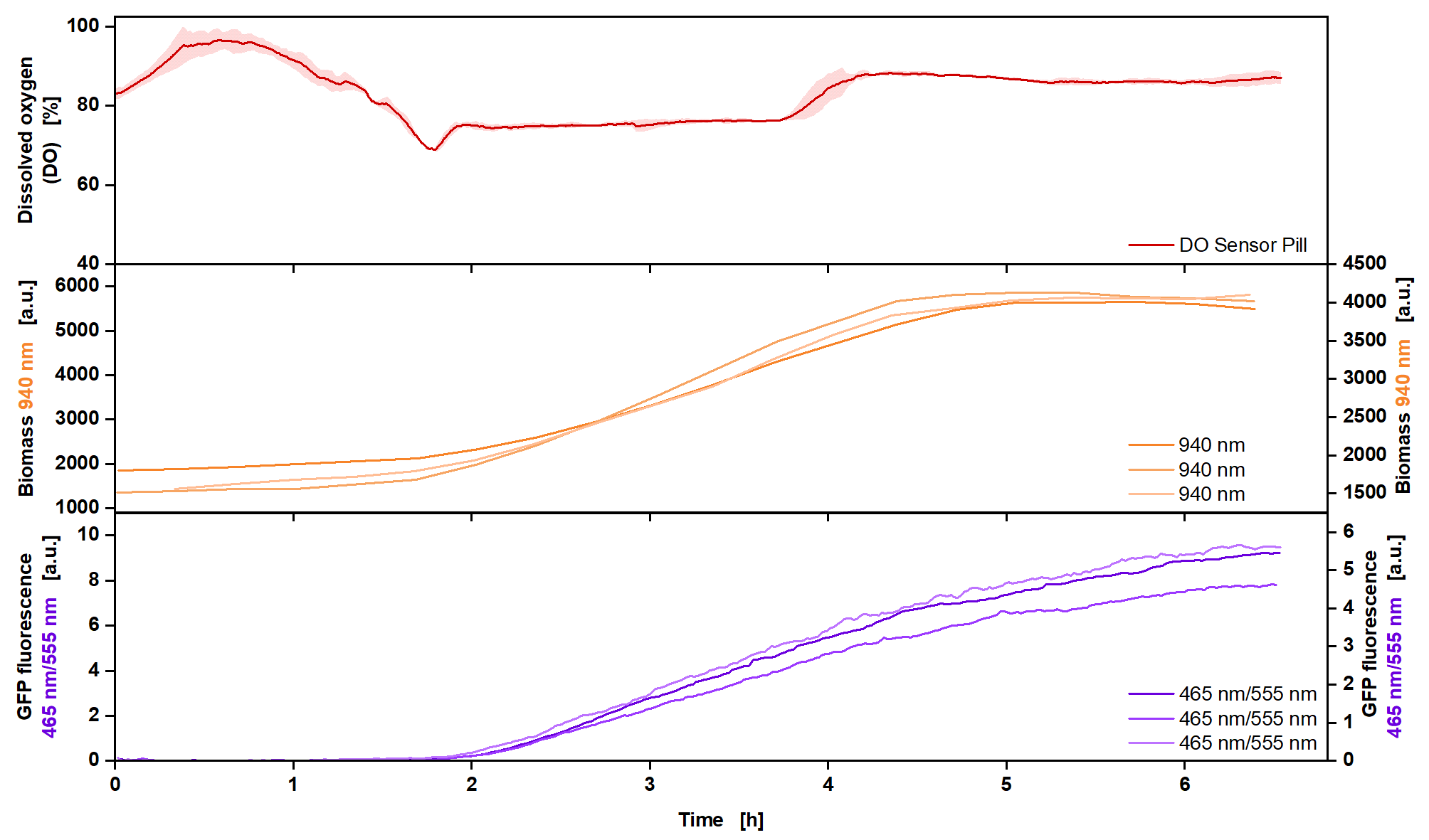


Figure S 13 Cultivation of Escherichia coli eGFP, LB-Kan medium, DO Sensor Pill (red) online biomass measurement at 940 nm (orange) and fluorescence measurement at 465 nm excitation and 555 nm emission (purple), V_L_ = 10%,(250 mL shaking flask), n = 250 rpm, d_0_ = 50 mm, t = 37 °C, rep. = 3, OD_600,Start_= 0.1. Shadows indicate the standard deviations.


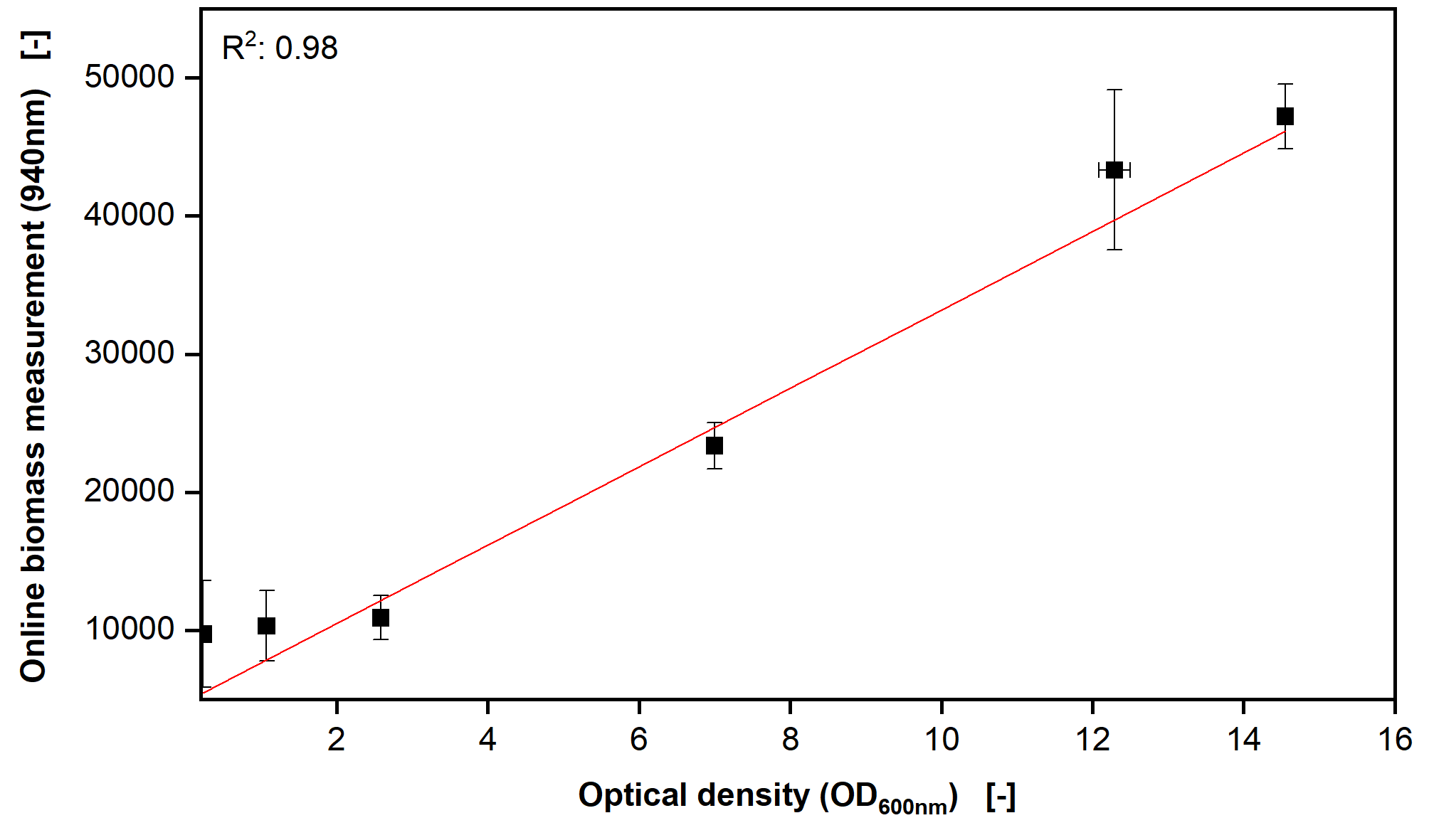


Figure S 14 Correlation between online measured backscatter at 940 nm and offline measured OD at 600 nm, cultivation with Corynebacterium glutamicum (Figure 6, main manuscript).


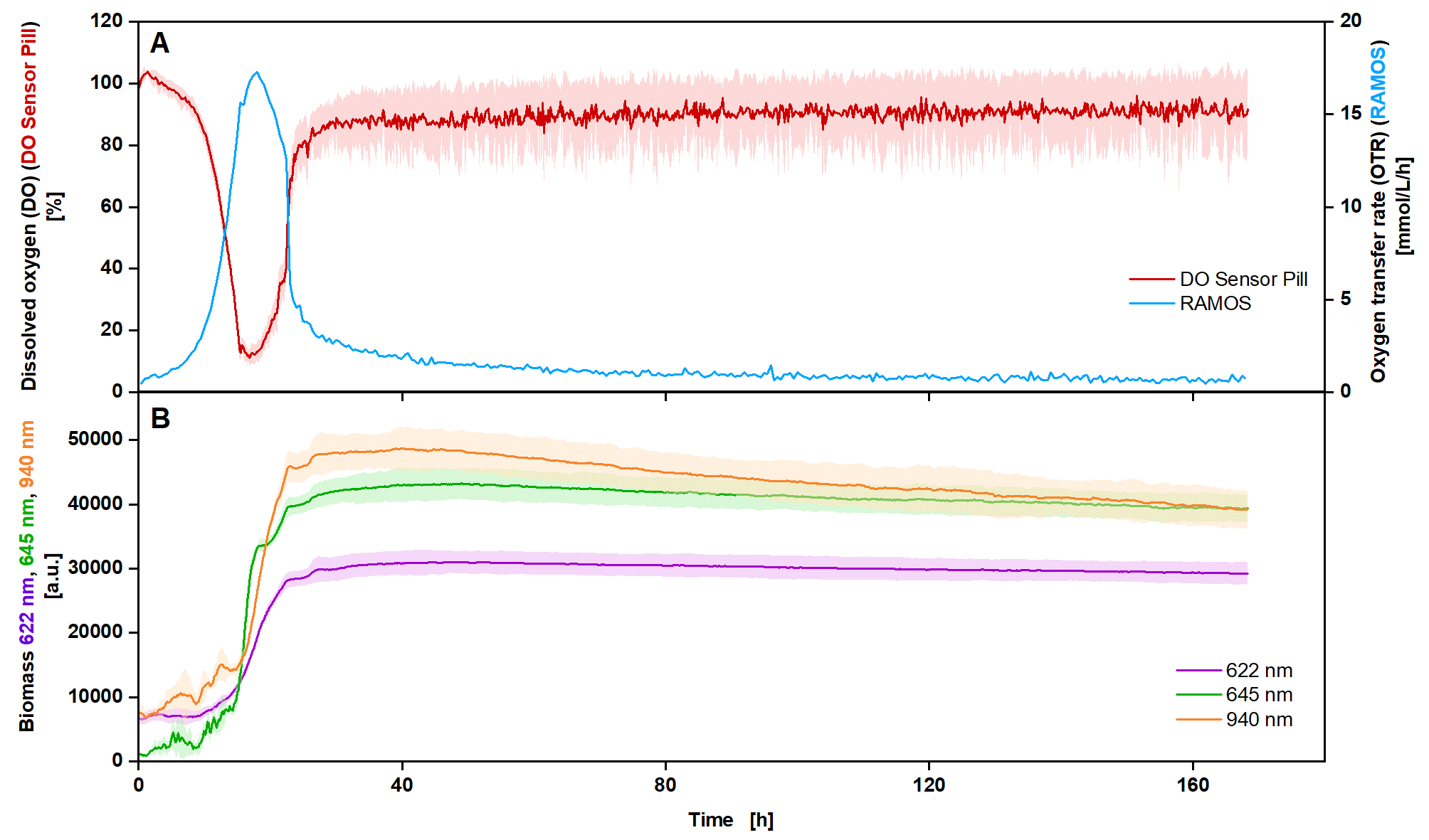


Figure S 15 Cultivation of Ustilago maydis MB215Δcyp1Δemt1, Verduny medium, V_L_= 10%, n= 250 rpm, d_0_= 50 mm, t= 30 °C, rep.= 3, OD_600_,_Start_= 0.1. OTR measurement via RAMOS (A, blue), DO Sensor Pill (A, red), biomass measurement at 622 nm (B, purple), 645 nm (B, green), 940 nm (B, orange). Shadows indicate the standard deviations.


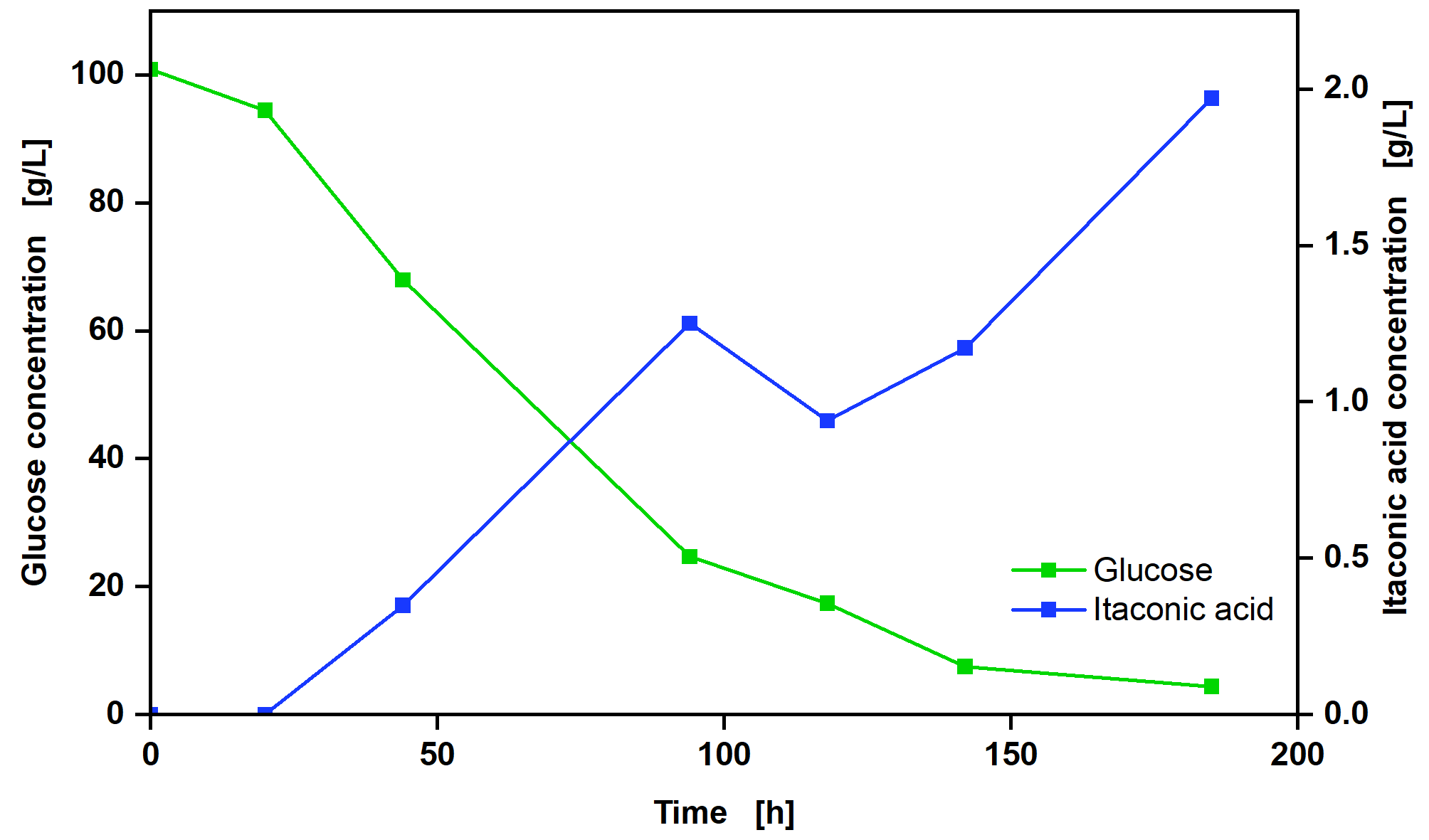


Figure S 16 Cultivation of Ustilago maydis MB215Δcyp1Δemt1, Verduyn medium, V_L_= 10%, n= 250 rpm, d_0_= 50 mm, t= 30 °C, rep.= 3, OD_600_,_Start_= 0.1. HPLC offline data, glucose (green), and itaconic acid (blue).


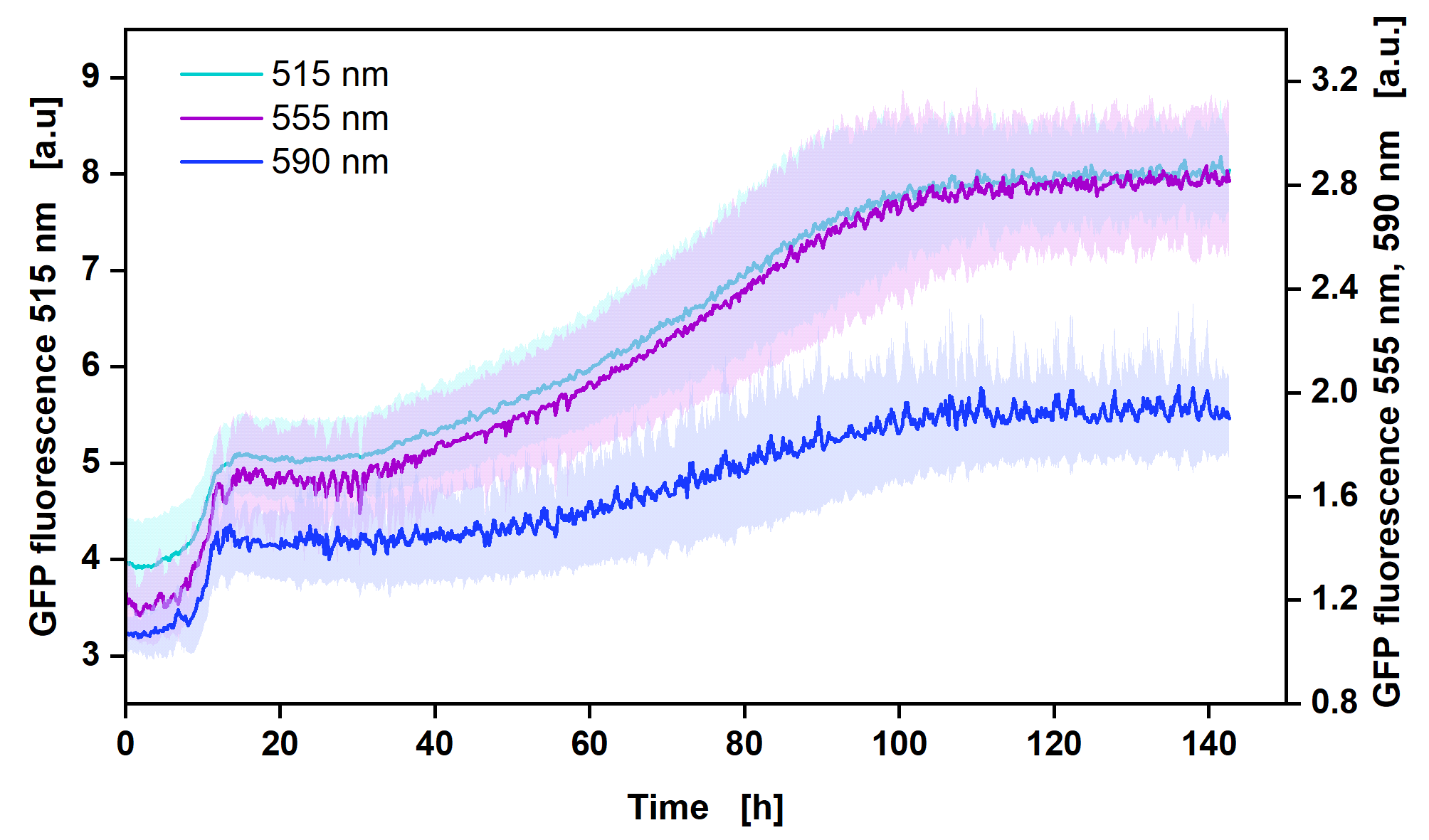


Figure S 17 Cultivation of Komagataella phaffii Mut^S^, Syn6 medium, online fluorescence measurement 465 nm excitation and emissions at 515 nm (turquoise), 555 nm (purple) and 590 nm(blue): V_L_= 10%, n= 350 rpm, d_0_= 50 mm, t= 30 °C, rep.= 3, OD_Start_= 0.2. Shadows indicate the standard deviations.
